# Completion rate and safety of non-invasive brain stimulation for upper limb dysfunction after stroke: a systematic review and network meta-analysis

**DOI:** 10.3389/fneur.2026.1853609

**Published:** 2026-09-09

**Authors:** Juan Sun, ZiLe Wang, Yiming Huang, Yilin Du, Sijia Zhang, Hui Shen, Huimin Zhang, Shuxian Feng

**Affiliations:** 1 Xinxiang Medical University, Xinxiang, China; 2 Xinxiang First People’s Hospital, Xinxiang, China

**Keywords:** network meta-analysis, non-invasive brain stimulation, repetitive transcranial magnetic stimulation, safety, stroke, transcranial direct current stimulation, treatment completion rate, upper limb

## Abstract

Non-invasive brain stimulation (NIBS), including repetitive transcranial magnetic stimulation (rTMS) and transcranial direct current stimulation (tDCS), has been widely used as an adjunctive intervention for upper limb motor recovery after stroke; however, the treatment completion rates and comparative safety profiles across different NIBS modalities have not been systematically evaluated. This systematic review and network meta-analysis aimed to assess and compare the treatment completion rates of various NIBS interventions for post-stroke upper limb rehabilitation. We systematically searched six databases and trial registries (PubMed, Embase, Cochrane CENTRAL, CINAHL, WHO ICTRP, and ClinicalTrials.gov) for randomized controlled trials (RCTs) published up to January 20, 2025, with an updated search conducted to April 15, 2026. Random-effects single-arm meta-analyses using the Freeman-Tukey double arcsine transformation were performed to pool completion rates, and a Bayesian random-effects network meta-analysis estimated relative risks (RRs) with 95% credible intervals (CrIs); treatments were ranked using surface under the cumulative ranking curve (SUCRA) values. Risk of bias was assessed using the Cochrane Risk of Bias 2 tool. The protocol was registered with PROSPERO (CRD420261309536). Fifty-five RCTs comprising 68 comparisons and 2,640 participants were included. The pooled intervention group completion rate was 94.9% (95% CI: 93.3–96.4%; I^2^ = 46.8%). The network meta-analysis showed no statistically significant differences in completion rate between any NIBS intervention and sham/control (all 95% CrIs included 1.00). SUCRA rankings indicated that intermittent theta burst stimulation (iTBS; 71.1%), bilateral rTMS (BL-rTMS; 60.9%), and dual transcranial direct current stimulation (Dual-tDCS; 58.1%) ranked highest. The overall adverse event (AE) rate was 16.4% (95% CI: 8.5–26.2%) in intervention groups, predominantly mild headache and skin tingling, with an AE-related dropout rate of only 1.1%. Subgroup analyses showed higher completion rates for tDCS (96.8%) than rTMS (93.9%), with the chronic phase highest (97.1%) and the mixed phase lowest (89.1%). The LFK index was 0.04, indicating no publication bias. In conclusion, NIBS for post-stroke upper limb rehabilitation demonstrates favorable tolerability and safety, with an overall completion rate of approximately 95%. Exploratory SUCRA rankings placed iTBS, BL-rTMS, and Dual-tDCS in the top three positions; however, these rankings should be considered hypothesis-generating rather than definitive, as four interventions were informed by only a single study, absolute differences in completion rates were minimal, and all pairwise credible intervals included 1.00. These observed phase-specific differences suggest that stroke phase may be an important factor to consider when designing adherence strategies, although prospective evaluation of tailored interventions is needed.

**Systematic review registration:**

https://www.crd.york.ac.uk/prospero/display_record.php?ID=CRD420261309536, PROSPERO (Registration ID: CRD420261309536).

## 1 Introduction

Stroke is the leading cause of long-term disability worldwide, with approximately 80 million stroke survivors globally as of 2023 (1). During the acute phase, 55–75% of stroke survivors experience upper limb motor dysfunction, and only a minority achieve full functional recovery within 6 months of onset (2, 3). Persistent upper limb impairment substantially compromises activities of daily living, quality of life, and social participation, while imposing a considerable economic burden on healthcare systems and patients’ families (4, 5). Despite advances in acute stroke management and rehabilitation, effective interventions to optimize upper limb recovery remain an important unmet clinical need.

Non-invasive brain stimulation (NIBS) has attracted growing attention as a promising adjunctive neuromodulatory intervention for promoting motor recovery after stroke (6, 7). NIBS primarily includes repetitive transcranial magnetic stimulation (rTMS) and transcranial direct current stimulation (tDCS), which modulate cortical excitability and promote neuroplasticity through distinct mechanisms (8). Although high-frequency rTMS and anodal tDCS are generally regarded as facilitatory, and low-frequency rTMS and cathodal tDCS as inhibitory, the directionality of these effects is increasingly recognized to be variable and dependent on individual and contextual factors (9–11).

The theoretical rationale for NIBS in stroke rehabilitation derives from the interhemispheric competition model, which posits that stroke-induced imbalance of interhemispheric inhibition contributes to motor deficits (12). Excitatory stimulation of the ipsilesional hemisphere or inhibitory stimulation of the contralesional hemisphere aims to restore interhemispheric balance and facilitate motor recovery (13). Randomized controlled trials (RCTs) and meta-analyses have demonstrated that NIBS combined with conventional rehabilitation improves upper limb motor function after stroke (14–16), with emerging modalities such as intermittent theta burst stimulation (iTBS) and dual transcranial direct current stimulation (Dual-tDCS) showing promising therapeutic potential (17–19).

Although efficacy outcomes have been extensively investigated, the treatment completion rates and safety profiles of different NIBS modalities have not been systematically examined. Treatment completion, defined as the proportion of enrolled participants who complete all planned intervention sessions, is a critical indicator for treatment tolerability, feasibility, and the potential for real-world clinical translation (20, 21). High dropout rates may reflect adverse effects, patient burden, logistical inconvenience, or intervention-related discomfort, thereby undermining the applicability of study findings to clinical practice (22). Understanding the factors associated with completion is therefore essential for designing implementable interventions, and differences in completion across modalities can help identify interventions that achieve an optimal balance between efficacy and tolerability (23). In addition, safety reporting in NIBS trials is frequently incomplete and lacks standardization, with inconsistent adverse-event definitions and monitoring procedures across studies (24, 25). Existing meta-analyses have largely relied on pairwise comparisons, which preclude the simultaneous ranking of multiple interventions (26), and completion and safety may further vary by stroke phase and treatment setting (27, 28). Network meta-analysis (NMA) provides a robust framework for simultaneously comparing multiple interventions and generating comprehensive rankings, even in the absence of direct head-to-head comparisons (29, 30).

Against this background, the objectives of the present systematic review and network meta-analysis were to: (1) comprehensively assess and compare the treatment completion rates of different NIBS modalities for upper limb rehabilitation in stroke patients; (2) rank all interventions by completion rate based on NMA results; (3) systematically evaluate the adverse event rates and safety profiles of each NIBS modality; (4) explore the influence of stimulation type, stroke phase, and treatment setting on completion rate heterogeneity through subgroup analyses and meta-regression; and (5) assess evidence quality and robustness of findings through sensitivity analyses.

## 2 Methods

### 2.1 Study design and registration

This systematic review and network meta-analysis was designed and reported in accordance with the Preferred Reporting Items for Systematic Reviews and Meta-Analyses extension for Network Meta-Analyses (PRISMA-NMA) (31). The protocol was prospectively registered in the International Prospective Register of Systematic Reviews (PROSPERO; registration number: CRD420261309536).

### 2.2 Search strategy

We systematically searched six sources—four bibliographic databases (PubMed, Embase, Cochrane CENTRAL, and CINAHL) and two trial registries (WHO ICTRP and ClinicalTrials.gov). Consistent with PRISMA-S guidance, the coverage of each source was: PubMed (1946–), Embase (1947–), Cochrane CENTRAL (1898–), CINAHL (1937–), WHO ICTRP (2007–), and ClinicalTrials.gov (2000–). The initial search was conducted on January 20, 2025. Because the manuscript was prepared approximately 15 months later, an updated search using the identical strategy was performed from January 21, 2025 to April 15, 2026 prior to submission. Searches were restricted to English-language publications. The strategy combined Medical Subject Headings (MeSH/Emtree) with free-text terms covering three domains: (1) population (stroke, cerebrovascular accident, hemiplegia, upper extremity); (2) intervention (transcranial magnetic stimulation, rTMS, theta burst stimulation, transcranial direct current stimulation, tDCS, non-invasive brain stimulation); and (3) study design (randomized controlled trial, RCT). The full PubMed strategy is provided in Supplementary Table S1, and strategies for other sources were adapted accordingly. Reference lists of included studies and relevant systematic reviews were manually screened.

### 2.3 Eligibility criteria

Studies were eligible if they met the following criteria: (1) participants: adults (≥18 years) with a clinical or imaging-confirmed diagnosis of stroke and upper limb dysfunction; (2) intervention: any form of NIBS, including rTMS and its subtypes (high-frequency rTMS, low-frequency rTMS, intermittent theta burst stimulation [iTBS], continuous theta burst stimulation [cTBS], bilateral rTMS, etc.) and tDCS and its subtypes (anodal tDCS, cathodal tDCS, dual tDCS, high-definition tDCS, etc.); (3) comparator: sham stimulation or conventional rehabilitation; (4) outcome: treatment completion rate or data from which it could be calculated (number enrolled and number completed); and (5) study design: RCT. Studies were excluded if they were non-randomized studies, case reports, reviews, or conference abstracts; animal or *in vitro* studies; or if the full text was unavailable or data were incomplete.

### 2.4 Study selection and data extraction

Two reviewers independently performed study selection and data extraction, with disagreements resolved by discussion or adjudication by a third reviewer. Screening was conducted in two stages: initial screening of titles and abstracts, followed by full-text assessment of potentially eligible articles. Data were extracted using a pre-designed standardized form, including: first author, publication year, country, sample size, stroke phase (acute ≤2 weeks, subacute 2 weeks to 6 months, chronic >6 months), intervention type and subtype, stimulation parameters, treatment setting, and the number of participants enrolled and completing the study in both intervention and control groups. Treatment completion was operationally defined as follows: by default, a participant was classified as a completer if they attended all planned NIBS sessions specified in the protocol (100% attendance); where a study reported a different threshold (e.g., ≥80% session attendance), the original definition was retained; and where the criterion was not explicitly stated, an intention-to-treat principle was applied (participants remaining in the final analysis sample were classified as completers). For non-completers, the reasons for discontinuation were extracted and categorized as lost to follow-up, adherence or personal reasons, disease-related causes, adverse-event-related withdrawal, or unspecified.

### 2.5 Risk of bias assessment

The risk of bias of included RCTs was assessed independently by two reviewers using the Cochrane Risk of Bias 2 (RoB 2) tool (32), across five domains (D1 randomization process; D2 deviations from intended interventions; D3 missing outcome data; D4 measurement of the outcome; D5 selection of the reported result), with each domain rated as low risk, some concerns, or high risk; disagreements were resolved by discussion.

### 2.6 Statistical analysis

Random-effects single-arm meta-analyses using the Freeman-Tukey double arcsine transformation were performed to pool completion rates with 95% confidence intervals (CIs) (33); between-study variance (*τ*^2^) was estimated using the DerSimonian-Laird method, and heterogeneity was assessed with Cochran’s Q test and the I^2^ statistic (*I*^2^ < 25% low, 25–50% moderate, >50% high) (34). For interventions represented by a single comparison (*k* = 1), 95% confidence intervals were calculated using the Clopper-Pearson exact method based on the binomial distribution.

A Bayesian random-effects NMA using sham/control as the reference estimated relative risks (RRs) and 95% credible intervals (CrIs), with treatments ranked using SUCRA and the probability of being best, P(Best) (35, 36). The evidence network had a star-shaped structure with no closed loops; formal inconsistency assessment was therefore not applicable, and transitivity was evaluated qualitatively by comparing baseline characteristics across studies.

Prespecified subgroup analyses were performed by stimulation type (rTMS vs. tDCS), stroke phase (acute, subacute, chronic, mixed), and treatment setting (inpatient, outpatient, laboratory, other), and multivariate meta-regression explored sources of heterogeneity (covariates: publication year, stimulation type, stroke phase). Session duration and total treatment span were not modeled because session duration was unreported in 23 of 55 studies (41.8%) and definitions varied among studies that did report it. Robustness was assessed through leave-one-out analyses, exclusion of studies at high risk of bias, and stratification by sample size, and through a sensitivity analysis excluding mixed-phase comparisons. Safety analysis pooled overall AE rates and AE-related dropout rates, stratified by AE type and stimulation type. Publication bias was assessed using the Doi plot and LFK index (|LFK| ≤ 1 indicating no asymmetry) (37), and evidence quality was appraised using the Grading of Recommendations, Assessment, Development and Evaluations (GRADE) framework.

All statistical analyses were performed using Python 3.8.16 and R version 4.3.1 (R Foundation for Statistical Computing, Vienna, Austria). Single-arm meta-analyses and meta-regression were conducted in Python using numpy (version 1.24.3), scipy (version 1.10.1), and pandas (version 2.0.3). Network meta-analysis was performed using the gemtc package (version 1.0–2) in R with JAGS version 4.3.1 for Bayesian Markov chain Monte Carlo inference. Forest plots, network diagrams, and other visualizations were generated using matplotlib (version 3.7.2) and seaborn (version 0.12.2).

## 3 Results

### 3.1 Study selection

**Figure 1:**
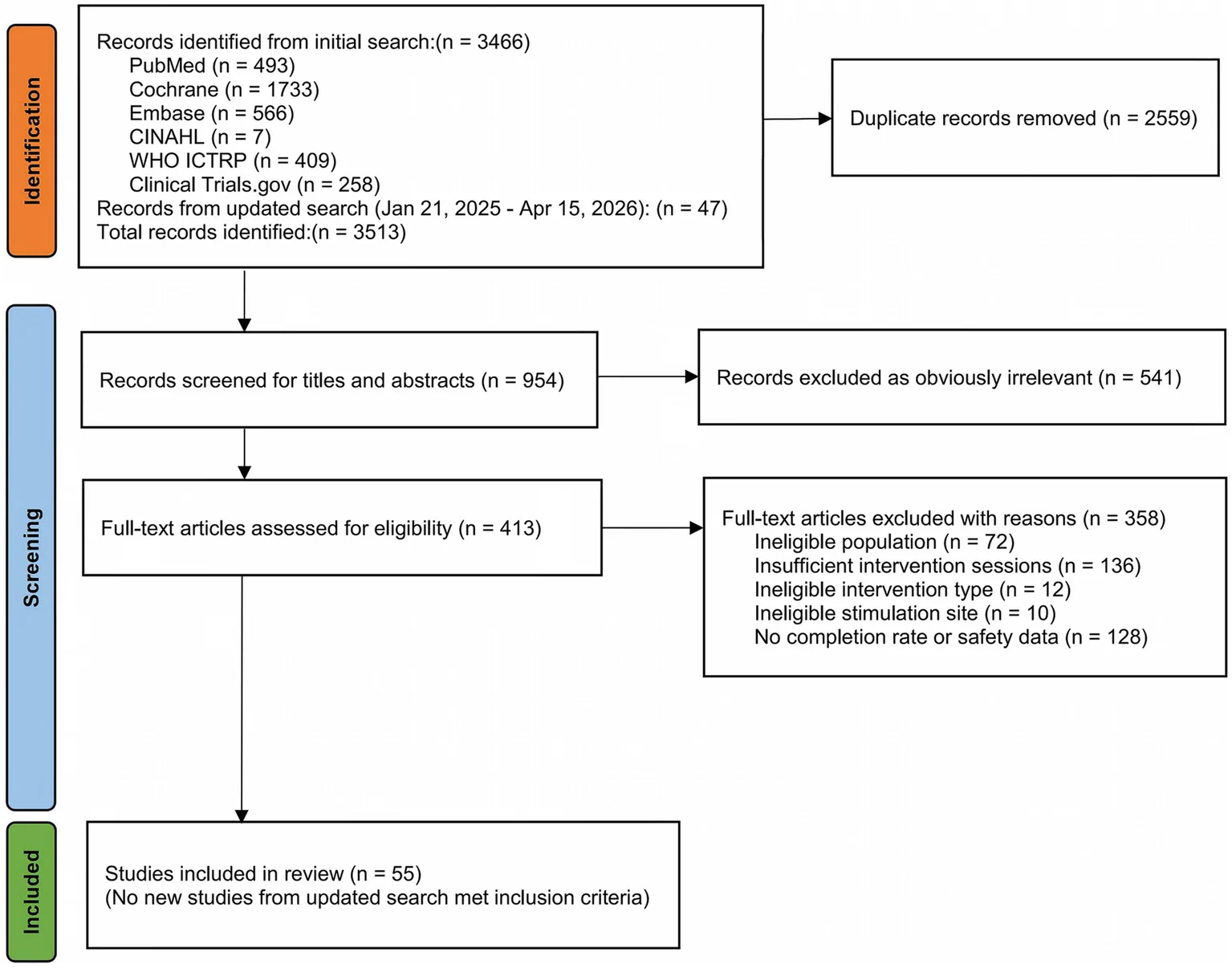
Study selection. PRISMA flow diagram of study identification, screening, and inclusion. Of 3,466 records identified and 954 screened after duplicate removal, 413 full-text articles were assessed and 55 RCTs were ultimately included.

Database searches yielded 3,466 records from the initial search (PubMed 493, Embase 566, Cochrane Library 1,733, CINAHL 7, WHO ICTRP 409, ClinicalTrials.gov 258) and 47 records from the updated search (January 21, 2025 to April 15, 2026), giving 3,513 total records. After 2,559 duplicate records were removed, 954 records were screened by title and abstract, of which 541 were excluded as obviously irrelevant. The full texts of 413 articles were assessed for eligibility, and 358 were excluded for the following reasons: inappropriate population (*n* = 72), inappropriate number of interventions (*n* = 136), interventions not rTMS/tDCS (*n* = 12), inappropriate intervention location (*n* = 10), and outcomes without completion-rate or safety data (*n* = 128). Ultimately, 55 RCTs comprising 68 independent comparisons were included in the quantitative synthesis (Figure 1). No records from the updated search met the inclusion criteria. The cumulative meta-analysis (Supplementary Figure S3C) and leave-one-out analyses confirmed that the primary completion-rate estimate was stable.

### 3.2 Study characteristics and network structure

The 55 included RCTs (38–92) were published between 2010 and 2025, spanning 17 countries and enrolling 2,640 participants (1,499 in intervention arms and 1,141 in control arms, after de-duplicating shared controls in multi-arm trials), with post-stroke upper limb dysfunction. rTMS-related studies contributed 43 comparisons (63.2%) and tDCS-related studies contributed 25 comparisons (36.8%). The distribution of intervention subtypes was LF-rTMS (15 comparisons), HF-rTMS (14), A-tDCS (12), Dual-tDCS (8), iTBS (4), BL-rTMS (3), BL-tDCS (2), Combined Protocol (2), cTBS (2), C-tDCS (2), BL-TBS (1), HD-tDCS (1), Priming iTBS (1), and Non-priming iTBS (1), totaling 68 comparisons. The stroke-phase distribution included subacute (25 comparisons, 36.8%), chronic (21, 30.9%), acute (13, 19.1%), and mixed (9, 13.2%), and treatment settings comprised inpatient (41, 60.3%), outpatient (16, 23.5%), laboratory (2, 2.9%), and other/not reported (9, 13.2%). Detailed study characteristics are presented in Table 1.

**Table 1:** Basic characteristics of eligible studies.

StudyID	Author	Year	Country	Type	Subtype	Phase	Int. Enrolled	Int. Completed	Con. Enrolled	Con. Completed	Int. CR (%)	Con. CR (%)	Setting	Ref
001	Harvey	2018	United States	rTMS	LF-rTMS	Subacute and chronic	132	124	67	63	93.9	94.0	Outpatient	(38)
002	Kim	2020	South Korea	rTMS	LF-rTMS	Subacute	40	36	37	37	90.0	100.0	Hospital	(39)
003	Chang	2010	South Korea	rTMS	HF-rTMS	Subacute	18	18	10	10	100.0	100.0	Inpatient	(40)
004	Viana	2014	Brazil	tDCS	A-tDCS	Chronic	10	10	10	10	100.0	100.0	Outpatient	(41)
005	Wang	2020	China	rTMS	HF-rTMS	Subacute	15	15	15	15	100.0	100.0	Inpatient	(42)
005	Wang	2020	China	rTMS	LF-rTMS	Subacute	15	15	15	15	100.0	100.0	Inpatient	(42)
006	Long	2018	China	rTMS	LF-rTMS	Acute	21	21	20	20	100.0	100.0	Inpatient	(43)
006	Long	2018	China	rTMS	BL-rTMS	Acute	21	21	20	20	100.0	100.0	Inpatient	(43)
007	Hsu	2023	Taiwan	tDCS	Dual-tDCS	Subacute	13	13	14	14	100.0	100.0	Inpatient	(44)
008	Ilić	2016	Serbia	tDCS	A-tDCS	Chronic	14	14	12	11	100.0	91.7	Outpatient	(45)
009	Guan	2017	China	rTMS	HF-rTMS	Acute	21	13	21	14	61.9	66.7	Inpatient	(46)
010	Lüdemann-Podubecká	2015	Germany	rTMS	LF-rTMS	Subacute	20	20	20	20	100.0	100.0	Inpatient	(47)
011	Tedesco Triccas	2015	United Kingdom	tDCS	A-tDCS	Mixed	12	11	11	11	91.7	100.0	Outpatient	(48)
012	Wang	2014	Taiwan	rTMS	LF-rTMS+iTBS	Subacute	17	17	16	16	100.0	100.0	Inpatient	(49)
012	Wang	2014	Taiwan	rTMS	iTBS+LF- rTMS	Subacute	15	15	16	16	100.0	100.0	Inpatient	(49)
013	Hosomi K	2016	Japan	rTMS	HF-rTMS	Subacute	20	18	21	21	90.0	100.0	Inpatient	(50)
014	Khan F	2019	India	rTMS	BL-TBS	Acute	20	20	20	20	100.0	100.0	Inpatient and outpatient	(51)
015	Hesse S	2011	Germany	tDCS	A-tDCS	Subacute	32	31	32	32	96.9	100.0	Inpatient	(52)
015	Hesse S	2011	Germany	tDCS	C-tDCS	Subacute	32	31	32	32	96.9	100.0	Inpatient	(52)
016	Unger RH	2023	United States	tDCS	A-tDCS	Chronic	11	9	14	11	81.8	78.6	Outpatient	(53)
017	Youssef H	2023	Egypt	tDCS	BL-tDCS	Subacute	11	11	11	11	100.0	100.0	Outpatient	(54)
017	Youssef H	2023	Egypt	tDCS	A-tDCS	Subacute	13	13	11	11	100.0	100.0	Outpatient	(54)
018	Zhang	2022	China	rTMS	Priming iTBS	Chronic	14	14	14	14	100.0	100.0	Outpatient	(55)
018	Zhang	2022	China	rTMS	Non-priming iTBS	Chronic	14	14	14	14	100.0	100.0	Outpatient	(55)
019	Conforto	2012	Brazil	rTMS	LF-rTMS	Subacute	15	15	15	14	100.0	93.3	Outpatient	(56)
020	Chervyakov	2018	Russia	rTMS	LF-rTMS	Subacute and chronic	18	11	11	10	61.1	90.9	Inpatient	(57)
020	Chervyakov	2018	Russia	rTMS	HF-rTMS	Subacute and chronic	18	13	11	10	72.2	90.9	Inpatient	(57)
021	Chen	2021	China	rTMS	BL-rTMS	Acute	32	31	32	30	96.9	93.8	Inpatient	(58)
021	Chen	2021	China	rTMS	HF-rTMS	Acute	32	25	32	25	78.1	78.1	Inpatient	(58)
022	Bornheim	2019	Belgium	tDCS	A-tDCS	Acute	25	25	25	25	100.0	100.0	Inpatient	(59)
023	Bolognini	2020	Italy	tDCS	Dual-tDCS	Acute	16	16	16	16	100.0	100.0	Inpatient	(60)
024	Straudi	2016	Italy	tDCS	Dual-tDCS	Mixed	12	12	11	11	100.0	100.0	NR	(61)
025	Seniów	2012	Poland	rTMS	LF-rTMS	Subacute	20	20	20	20	100.0	100.0	Inpatient	(62)
026	Mazzoleni	2019	Italy	tDCS	A-tDCS	Subacute	20	20	20	19	100.0	95.0	Inpatient	(63)
027	Etoh	2013	Japan	rTMS	LF-rTMS	Chronic	18	18	18	18	100.0	100.0	NR	(64)
028	Emara	2010	Egypt	rTMS	HF-rTMS	Chronic	20	20	20	20	100.0	100.0	NR	(65)
028	Emara	2010	Egypt	rTMS	LF-rTMS	Chronic	20	20	20	20	100.0	100.0	NR	(65)
029	Shim	2023	South Korea	rTMS	HF-rTMS	Subacute	17	14	16	16	82.4	100.0	Inpatient	(66)
030	Di Lazzaro	2016	Italy	rTMS	cTBS	Chronic	10	8	10	9	80.0	90.0	Outpatient	(67)
031	Lai	2015	Taiwan	rTMS	iTBS	Chronic	21	21	17	17	100.0	100.0	Inpatient	(68)
032	Liao	2025	Taiwan	tDCS	A-tDCS	Chronic	12	12	12	12	100.0	100.0	Hospital	(69)
032	Liao	2025	Taiwan	tDCS	A-tDCS	Chronic	12	12	12	12	100.0	100.0	Hospital	(69)
033	Rose	2014	United States	rTMS	LF-rTMS	Chronic	11	9	10	10	81.8	100.0	Outpatient	(70)
034	Moslemi Haghighi	2021	Iran	rTMS	HF-rTMS	Subacute	10	10	10	10	100.0	100.0	NR	(71)
035	Ke	2020	China	rTMS	HF-rTMS	Acute	16	14	16	15	87.5	93.8	Inpatient	(72)
035	Ke	2020	China	rTMS	HF-rTMS	Acute	16	15	16	15	93.8	93.8	Inpatient	(72)
036	Shaheiwola	2018	China	tDCS	BL-tDCS	Chronic	15	15	15	15	100.0	100.0	Hospital	(73)
037	Ni	2023	China	rTMS	HF-rTMS	Subacute	18	18	18	15	100.0	83.3	Inpatient	(74)
038	Chen	2025	China	rTMS	iTBS	Subacute	25	25	25	23	100.0	92.0	Inpatient	(75)
039	Wang	2023	China	rTMS	LF-rTMS	Chronic	23	23	23	23	100.0	100.0	Inpatient	(76)
040	Wu	2023	China	rTMS	HF-rTMS	Subacute	15	15	15	15	100.0	100.0	Inpatient	(77)
041	Yang	2021	China	rTMS	HF-rTMS	Subacute	14	12	15	13	85.7	86.7	Inpatient and outpatient	(78)
042	Edwards	2018	United States	tDCS	A-tDCS	Chronic	41	40	41	37	97.6	90.2	Outpatient	(79)
043	Palimeris	2022	Canada	tDCS	A-tDCS	Chronic	48	48	42	42	100.0	100.0	Outpatient	(80)
044	Liu	2025	China	rTMS	LF-rTMS	Subacute	35	34	35	30	97.1	85.7	Inpatient	(81)
045	Chen	2021	China	rTMS	BL-rTMS	Acute	24	22	24	22	91.7	91.7	Inpatient	(82)
046	Gao	2025	China	tDCS	Dual-tDCS	Subacute	22	20	23	19	90.9	82.6	Inpatient	(83)
047	Tang	2024	China	rTMS	iTBS	Mixed	15	15	15	14	100.0	93.3	Inpatient	(84)
047	Tang	2024	China	rTMS	cTBS	Mixed	15	14	15	14	93.3	93.3	Inpatient	(84)
048	Morone	2022	Italy	tDCS	Dual-tDCS	Chronic	33	33	33	33	100.0	100.0	Outpatient	(85)
049	Lee SH	2025	South Korea	tDCS	HD-tDCS	Chronic	14	12	14	12	85.7	85.7	Outpatient	(86)
050	Chen	2019	Taiwan	rTMS	iTBS	Chronic	12	11	11	11	91.7	100.0	Inpatient	(87)
051	Salazar	2020	Brazil	tDCS	Dual-tDCS	Chronic	15	15	15	15	100.0	100.0	Outpatient	(88)
052	Pinto	2021	India	tDCS	Dual-tDCS	Mixed	31	29	29	24	93.5	82.8	Inpatient	(89)
053	Zheng	2015	China	rTMS	LF-rTMS	Subacute	58	55	54	53	94.8	98.1	Inpatient	(90)
054	Zhao	2022	China	tDCS	Dual-tDCS	Acute	30	30	30	30	100.0	100.0	Inpatient	(91)
055	Yao	2020	China	tDCS	C-tDCS	Subacute and chronic	22	20	20	20	90.9	100.0	Inpatient	(92)

Int., Intervention; Con., Control; CR, Completion Rate (%); Type, Intervention type (rTMS or tDCS); Subtype, Intervention subtype; Phase, Stroke phase; *N*, number of participants; NR, Not Reported; Ref, Reference number.

**Figure 2:**
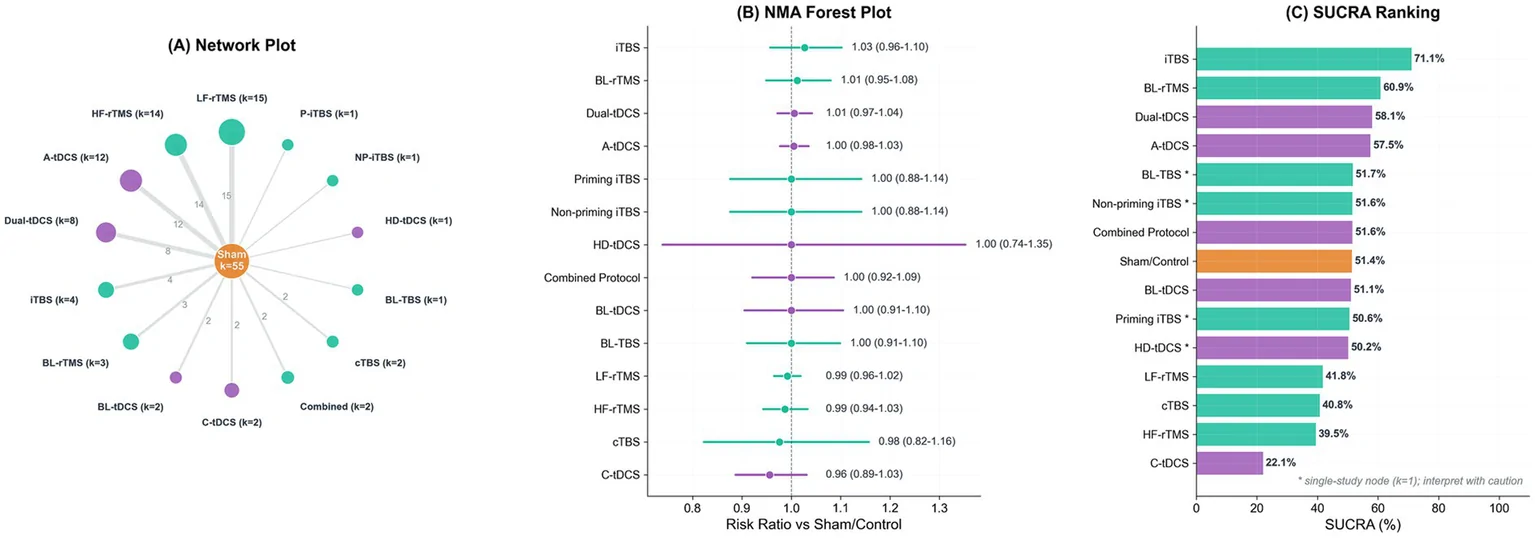
Network meta-analysis results. **(A)** Network plot (node size proportional to sample size; edge width proportional to the number of comparisons); **(B)** Forest plot of risk ratios versus sham/control; **(C)** SUCRA ranking. Higher SUCRA values indicate higher probability of ranking first. Interventions marked with asterisks (*) are based on single studies (*k* = 1) and should be interpreted with caution due to wide credible intervals. Note that all 95% credible intervals included 1.00, indicating no statistically significant differences between interventions.

The evidence network exhibited a purely star-shaped structure (Figure 2A), comprising 14 NIBS interventions and one control node, for a total of 15 nodes; all interventions were connected exclusively through the sham/control node, with no closed loops. Node size and evidence volume varied considerably: LF-rTMS (15 comparisons) and HF-rTMS (14 comparisons) represented the most information-rich nodes, whereas BL-TBS, HD-tDCS, Priming iTBS, and Non-priming iTBS each contributed only one comparison.

### 3.3 Risk of bias

**Figure 3:**
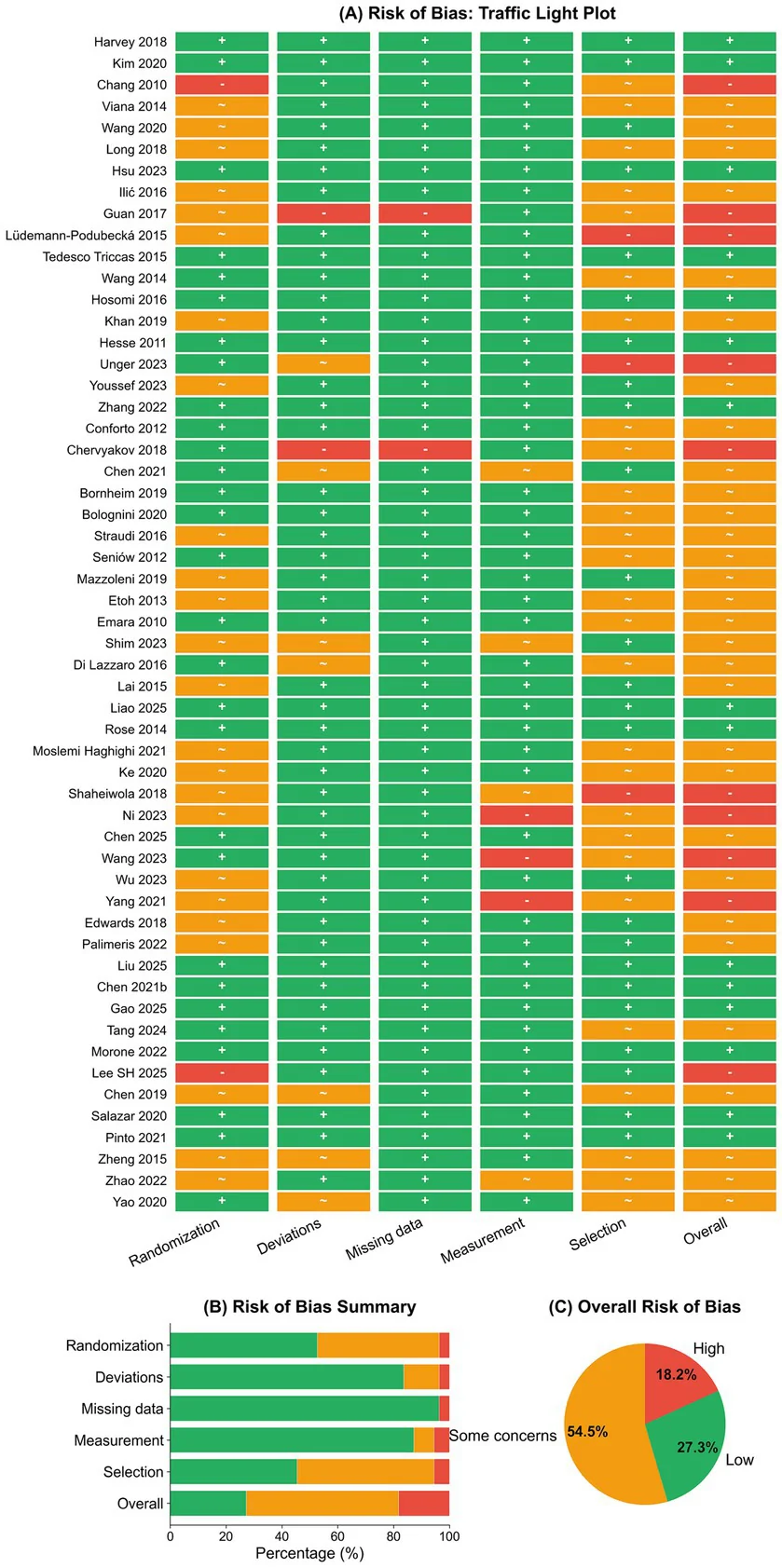
Risk of bias assessment (RoB 2). **(A)** Domain-level traffic-light plot for each study; **(B)** Summary of risk-of-bias proportions by domain; **(C)** Overall risk-of-bias distribution.

Overall risk of bias was rated as low in 15 studies (27.3%), some concerns in 30 (54.5%), and high in 10 (18.2%) (Figure 3). High risk of bias was primarily attributable to inadequate randomization procedures and selective reporting (Supplementary Table S4).

### 3.4 Treatment completion rate

#### 3.4.1 Overall completion rate

The pooled intervention group completion rate was 94.9% (95% CI: 93.3–96.4%; *I*^2^ = 46.8%; *k* = 68), and the pooled control group completion rate was 95.3% (95% CI: 93.8–96.7%), suggesting that NIBS did not contribute to additional dropout (Supplementary Table S2).

#### 3.4.2 Completion rate by intervention subtype

**Figure 4:**
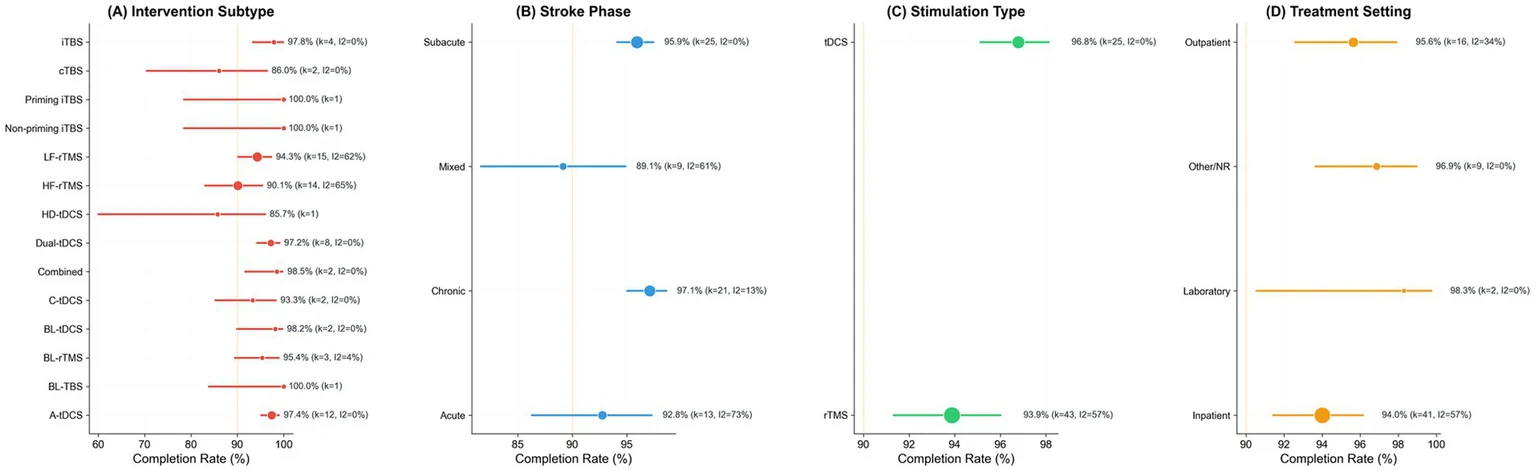
Subgroup analyses of completion rate (pooled rate with 95% CI): **(A)** By intervention subtype, **(B)** By stroke phase, **(C)** By stimulation type, **(D)** By treatment setting.

Completion rates varied across the 14 subtypes (Table 2; Figure 4A). Among subtypes with multiple comparisons, the highest rates were observed for Combined Protocol (98.5%; k = 2), BL-tDCS (98.2%; k = 2), iTBS (97.8%; k = 4), and A-tDCS (97.4%; k = 12), whereas HF-rTMS (90.1%; k = 14; I^2^ = 64.8%) and cTBS (86.0%; k = 2) showed relatively lower rates. The single-comparison subtypes (BL-TBS, Priming iTBS, and Non-priming iTBS; each 100.0%, k = 1) should be interpreted with caution.

**Table 2:** Completion by intervention.

Intervention	*k*	Pooled rate (%)	95% CI	*I*^**2**^ (%)	*p* for Q
A-tDCS	12	97.4	[95.1, 99.0]	0.0	0.637
BL-TBS	1	100.0	[83.9, 100.0]	0.0	1.000
BL-rTMS	3	95.4	[89.5, 98.9]	3.9	0.353
BL-tDCS	2	98.2	[89.9, 99.7]	0.0	0.918
C-tDCS	2	93.3	[85.3, 98.3]	0.0	0.371
Combined	2	98.5	[91.7, 99.8]	0.0	0.966
Dual-tDCS	8	97.2	[94.3, 99.1]	0.0	0.528
HD-tDCS	1	85.7	[60.1, 96.0]	0.0	1.000
HF-rTMS	14	90.1	[83.1, 95.4]	64.8	<0.001
LF-rTMS	15	94.3	[90.1, 97.3]	62.4	<0.001
Non-priming iTBS	1	100.0	[78.5, 100.0]	0.0	1.000
Priming iTBS	1	100.0	[78.5, 100.0]	0.0	1.000
cTBS	2	86.0	[70.4, 96.4]	0.0	0.347
iTBS	4	97.8	[93.4, 99.9]	0.0	0.512

k, Number of comparisons; Pooled Rate, Pooled completion rate estimated using random-effects meta-analysis; CI, Confidence interval; *I*^2^, heterogeneity statistic; *p* for Q, *p*-value for Cochran’s Q test of heterogeneity. Combined = LF-rTMS + iTBS or iTBS + LF-rTMS sequential protocol.

#### 3.4.3 Reasons for dropout

Among studies reporting attrition data, the most common reasons for dropout included lost to follow-up, adherence or personal reasons, disease-related causes, and adverse event-related withdrawal: most studies reported a total dropout of ≤5 participants. Notably, none of the included studies explicitly documented treatment discontinuation due to sufficient functional recovery or early discharge, and the predefined categories did not include a “recovery / no longer indicated” category; it is therefore unclear whether such cases were absent or simply not captured (Supplementary Table S3; Supplementary Figure S2A).

### 3.5 Network meta-analysis

#### 3.5.1 Relative effect estimates

With sham/control as the reference, none of the 14 NIBS interventions differed significantly from control in completion rate (all 95% CrIs included 1.00) (Table 3; Figure 2B). RRs were highest for iTBS (1.027; 95% CrI: 0.957–1.102), BL-rTMS (1.012; 0.949–1.080), and Dual-tDCS (1.006; 0.972–1.042), and lowest for C-tDCS (0.956; 0.887–1.031); none reached statistical significance (Supplementary Table S8; Supplementary Figure S1B).

**Table 3:** Network meta-analysis results: Relative risk of completion rate (vs sham/control).

Treatment	k	RR	95% CrI	*I*^**2**^ (%)
A-tDCS	12	1.005	[0.977, 1.035]	0.0
BL-TBS	1	1.000	[0.910, 1.099]	0.0
BL-rTMS	3	1.012	[0.949, 1.080]	0.0
BL-tDCS	2	1.000	[0.905, 1.105]	0.0
C-tDCS	2	0.956	[0.887, 1.031]	0.0
Combined Protocol	2	1.000	[0.921, 1.086]	0.0
Dual-tDCS	8	1.006	[0.972, 1.042]	0.0
HD-tDCS	1	1.000	[0.739, 1.353]	0.0
HF-rTMS	14	0.987	[0.943, 1.033]	0.0
LF-rTMS	15	0.992	[0.965, 1.019]	0.0
Non-priming iTBS	1	1.000	[0.876, 1.142]	0.0
Priming iTBS	1	1.000	[0.876, 1.142]	0.0
cTBS	2	0.976	[0.823, 1.157]	0.0
iTBS	4	1.027	[0.957, 1.102]	0.0

RR, Relative risk; CrI, Credible interval; k, Number of comparisons. Reference group: Sham/Control. RR > 1.00 indicates higher completion rate compared with Sham/Control. All 95% CrIs include 1.00, indicating no statistically significant differences in completion rate between any NIBS intervention and Sham/Control. Combined Protocol = LF-rTMS + iTBS or iTBS + LF-rTMS sequential protocol.

#### 3.5.2 Inconsistency assessment

The network had a purely star-shaped structure, with no closed loops; node-splitting was therefore structurally inapplicable, and the consistency assumption could not be tested quantitatively. The plausibility of the transitivity assumption was evaluated qualitatively by comparing the similarity of baseline characteristics (stroke phase, treatment setting) across studies of different intervention subtypes.

#### 3.5.3 Treatment rankings

SUCRA analysis (Table 4; Figure 2C; Supplementary Figure S1A) ranked the top three interventions as iTBS (SUCRA = 71.1%), BL-rTMS (SUCRA = 60.9%), and Dual-tDCS (SUCRA = 58.1%). These rankings should be interpreted with substantial caution. First, absolute differences were minimal and all pairwise 95% CrIs included 1.00. Second, four interventions (BL-TBS, HD-tDCS, Priming iTBS, Non-priming iTBS) were informed by only a single study, and four others by only two, yielding wide credible intervals. Third, the discordance between SUCRA and P(Best) illustrates this instability: HD-tDCS ranked 11th yet had the highest P(Best) of 29.5%, reflecting the extreme uncertainty of a single small study with a very wide CrI (0.739–1.353), whereas top-ranked iTBS had a P(Best) of only 11.6%. These rankings should therefore be regarded as exploratory and hypothesis-generating rather than definitive evidence of superiority.

**Table 4:** SUCRA ranking of interventions for completion rate.

Rank	Treatment	SUCRA (%)	Mean Rank	P (Best) (%)	*k*
1	iTBS	71.1	5.0	11.6	4
2	BL-rTMS	60.9	6.5	4.9	3
3	Dual-tDCS	58.1	6.9	0.8	8
4	A-tDCS	57.5	6.9	0.4	12
5	BL-TBS*	51.7	7.8	6.2	1
6	Combined Protocol	51.6	7.8	4.8	2
7	Non-priming iTBS*	51.6	7.8	12.4	1
8	Sham/Control	51.4	7.8	0.0	68
9	BL-tDCS	51.1	7.8	6.7	2
10	Priming iTBS*	50.6	7.9	11.6	1
11	HD-tDCS*	50.2	8.0	29.5	1
12	LF-rTMS	41.8	9.1	0.1	15
13	cTBS	40.8	9.3	10.5	2
14	HF-rTMS	39.5	9.5	0.3	14
15	C-tDCS	22.1	11.9	0.2	2

SUCRA, Surface under the cumulative ranking curve; a higher SUCRA value indicates a higher probability of being the best intervention for completion rate. Mean Rank = average ranking across all simulations. *p* (Best) = probability of being ranked as the best treatment. k = number of comparisons contributing to each intervention. Rankings are based on Bayesian network meta-analysis. Combined Protocol = LF-rTMS + iTBS or iTBS + LF-rTMS sequential protocol. *Single-study interventions (k = 1) should be interpreted with substantial caution due to wide credible intervals and extreme sensitivity to study-specific characteristics. Note the discordance between SUCRA rankings and *p* (Best) values for single-study interventions: HD-tDCS ranked 11th but had the highest *p* (Best) (29.5%), reflecting the wide uncertainty from a single small study with a credible interval of [0.74, 1.35].

### 3.6 Safety analysis

**Figure 5:**
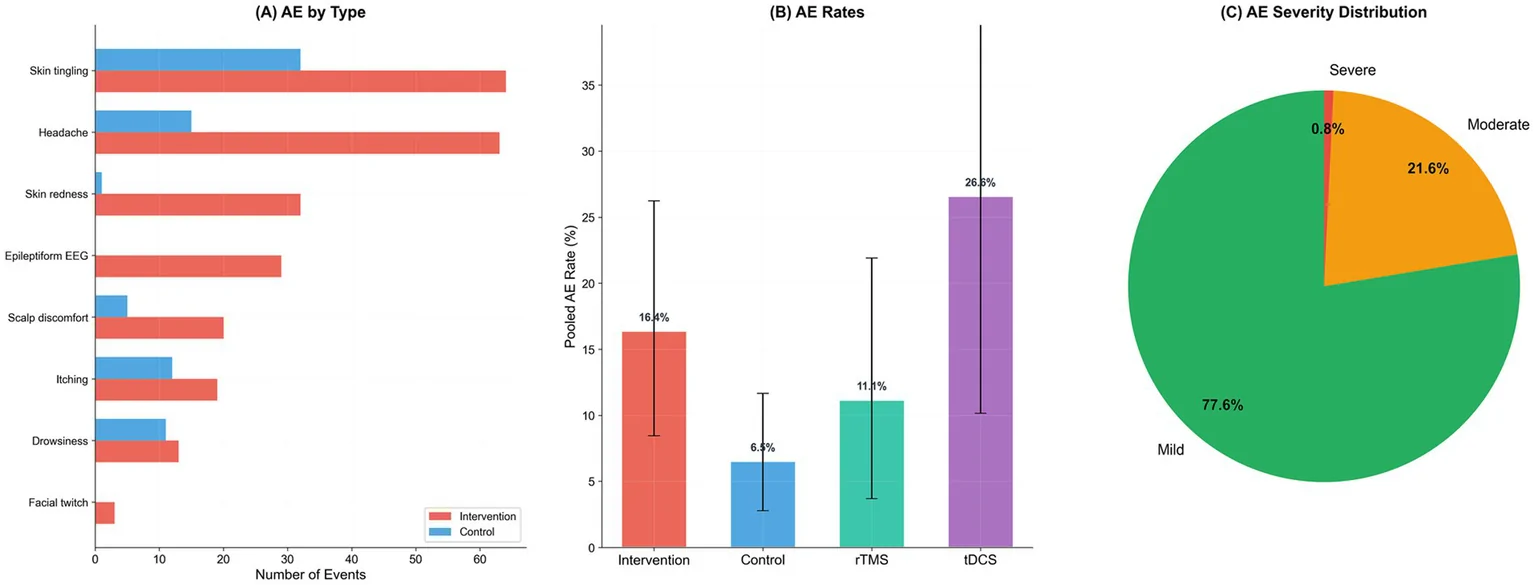
Safety profile **(A)** Adverse events by type in intervention and control groups; **(B)** Pooled AE rates by group and stimulation type; **(C)** AE severity distribution.

Safety data were analyzed from 42 studies reporting AEs (Figure 5B). The pooled overall AE rate was 16.4% (95% CI: 8.5–26.2%; *I*^2^ = 94.2%) in intervention groups and 6.5% (95% CI: 2.8–11.7%; *I*^2^ = 86.1%) in control groups. By stimulation type, the AE rate was 11.1% (95% CI: 3.7–21.9%; k = 26) for rTMS and 26.6% (95% CI: 10.2–47.3%; k = 16) for tDCS, and AE-related dropout was low and comparable between groups (intervention: 1.1%; control: 1.1%). The most frequently reported AEs were skin tingling (12 studies; 64 events) and headache (12 studies; 63 events), followed by skin redness (32 events) (Figure 5A); the vast majority were mild and self-resolving (Supplementary Table S10; Figure 5C), and only one study (Chervyakov 2018) reported serious adverse events (two seizures in the rTMS group, both requiring medical treatment and leading to withdrawal). This substantial heterogeneity in AE rates (*I*^2^ = 94.2%) most likely reflects between-study differences in AE definitions, monitoring protocols (structured checklists versus spontaneous reporting), and reporting thresholds, rather than true variation in safety profiles.

### 3.7 Subgroup analyses

Prespecified subgroup analyses yielded the following results (Table 5; Figure 4): By stimulation type: tDCS showed a higher pooled completion rate (96.8%; 95% CI: 95.1–98.1%; k = 25; *I*^2^ = 0.0%), than rTMS (93.9%; 95% CI: 91.3–96.0%; k = 43; *I*^2^ = 56.8%) (Figure 4C). By stroke phase: Chronic phase had the highest completion rate (97.1%; k = 21), followed by subacute (95.9%; k = 25) and acute (92.8%; k = 13), with the mixed phase lowest (89.1%; k = 9). Both the acute and mixed phase subgroups showed substantial heterogeneity (*I*^2^ = 72.6 and 60.6%, respectively) (Figure 4B); the high heterogeneity in the mixed phase subgroup suggests that this category may aggregate studies with diverse populations and protocols, and the associated estimate should be interpreted cautiously, as it may reflect study-level rather than true patient-level effects. By treatment setting: completion rates were comparable across inpatient (94.0%; k = 41), outpatient (95.6%; k = 16), laboratory (98.3%; k = 2), and other/not reported (96.9%; k = 9) settings (Figure 4D; Table 5).

**Table 5:** Subgroup analysis and meta-regression of intervention group completion rate.

Subgroup	Level	k	Pooled Rate (%)	95% CI	*I*^**2**^ (%)	*p* for Q
Stimulation type						
	rTMS	43	93.9	[91.3, 96.0]	56.8	<0.001
	tDCS	25	96.8	[95.1, 98.1]	0.0	0.628
Intervention subtype						
	A-tDCS	12	97.4	[95.1, 99.0]	0.0	0.637
	BL-TBS	1	100.0	[83.9, 100.0]	0.0	1.000
	BL-rTMS	3	95.4	[89.5, 98.9]	3.9	0.353
	BL-tDCS	2	98.2	[89.9, 99.7]	0.0	0.918
	C-tDCS	2	93.3	[85.3, 98.3]	0.0	0.371
	Combined	2	98.5	[91.7, 99.8]	0.0	0.966
	Dual-tDCS	8	97.2	[94.3, 99.1]	0.0	0.528
	HD-tDCS	1	85.7	[60.1, 96.0]	0.0	1.000
	HF-rTMS	14	90.1	[83.1, 95.4]	64.8	<0.001
	LF-rTMS	15	94.3	[90.1, 97.3]	62.4	0.001
	Non-priming iTBS	1	100.0	[78.5, 100.0]	0.0	1.000
	Priming iTBS	1	100.0	[78.5, 100.0]	0.0	1.000
	cTBS	2	86.0	[70.4, 96.4]	0.0	0.347
	iTBS	4	97.8	[93.4, 99.9]	0.0	0.512
Stroke phase						
	Acute	13	92.8	[86.3, 97.3]	72.6	<0.001
	Subacute	25	95.9	[94.1, 97.4]	0.0	0.621
	Chronic	21	97.1	[95.0, 98.6]	12.8	0.292
	Mixed	9	89.1	[81.6, 94.9]	60.6	0.009
Treatment setting						
	Inpatient	41	94.0	[91.4, 96.1]	56.8	<0.001
	Outpatient	16	95.6	[92.6, 97.9]	34.3	0.088
	Laboratory	2	98.3	[90.5, 99.7]	0.0	1.000
	Other/NR	9	96.9	[93.6, 99.0]	0.0	0.697

Subgroup	Level	*β*	SE	*p*
Meta-regression (multivariate)ᵃ				
Intercept	—	377.310	483.798	0.439
Publication year	—	−0.139	0.240	0.563
Stimulation type	tDCS (ref: rTMS)	3.577	2.151	0.101
Stroke phase	Acute (ref: Subacute)	−5.263	2.779	0.063
	Subacute+Chronic	−17.343	4.348	<0.001***
	Chronic	−1.722	2.446	0.484
	Mixed	−2.222	4.019	0.582
			

k, Number of comparisons; CI, Confidence interval; *I*^2^, heterogeneity statistic; *p* for Q, *p*-value for Cochran’s Q test of heterogeneity. Pooled rates were estimated using random-effects meta-analysis with Freeman-Tukey double arcsine transformation. *β*, regression coefficient; SE, standard error. ᵃ Multivariate random-effects meta-regression with publication year (mean-centered at 2018), stimulation type, and stroke phase as covariates. The dependent variable is completion rate (%). Subacute phase serves as the reference group for stroke phase; rTMS serves as the reference group for stimulation type. ***p < 0.001.

### 3.8 Meta-regression

Multivariate meta-regression (Table 5) identified “subacute + chronic” (mixed phase) as a negative predictor of completion rate (*β* = −17.343, *p* < 0.001), with acute phase showing a borderline trend (*β* = −5.263, *p* = 0.063); publication year (*β* = −0.139, *p* = 0.563) and stimulation type (*β* = 3.577, *p* = 0.101) were not significant. Univariate analyses confirmed that publication year (*β* = −0.097, *p* = 0.712), intervention group sample size (*β* = −0.006, *p* = 0.932), and number of planned sessions (*β* = 0.119, *p* = 0.460) were not significantly associated with completion rate (Supplementary Table S9). The lower completion observed in the mixed-phase subgroup should be interpreted cautiously, because this category aggregates trials spanning multiple stroke stages and exhibited high within-group heterogeneity (*I*^2^ = 60.6%); moreover, the number of planned sessions does not capture total treatment burden, which may represent an important unmeasured confounder.

### 3.9 Sensitivity analyses

Multiple sensitivity analyses confirmed the robustness of the findings. Leave-one-out analysis yielded pooled completion rates ranging from 94.8 to 95.2%; excluding 10 high-risk-of-bias studies gave 95.7% (95% CI: 94.3–96.9%); and stratification by sample size gave consistent results (large studies, *n* ≥ 30: 94.3%; small studies, *n* < 30: 95.2%). In a sensitivity analysis excluding the 9 mixed-phase comparisons, the pooled completion rate was 95.7% (95% CI: 94.1–97.0%; *I*^2^ = 40.1%), essentially unchanged from the overall estimate of 94.9% and confirming that the mixed-phase category did not materially influence the primary finding (Supplementary Table S6; Supplementary Figure S3A).

### 3.10 Publication bias

The Doi plot and LFK index yielded an LFK value of 0.04 (|LFK| ≤ 1), indicating no evidence of publication bias. No significant difference in completion rates was observed between early studies (pre-2018; 95.2%) and recent (2018 onwards; 94.2%) studies, and the cumulative meta-analysis showed that the pooled estimate stabilized as studies accrued (Supplementary Figures S2B, S3).

### 3.11 Quality of evidence

Using GRADE, evidence quality was rated moderate for A-tDCS, HF-rTMS, and LF-rTMS (downgraded one level for risk of bias), very low for HD-tDCS (downgraded for risk of bias, imprecision, and publication bias), and low for the remaining comparisons (Supplementary Table S13).

## 4 Discussion

Based on 55 RCTs comprising 68 comparisons and 2,640 participants, this network meta-analysis provides the most comprehensive synthesis to date of treatment completion rates and safety profiles of NIBS for post-stroke upper limb rehabilitation. The overall completion rate of approximately 95% indicates that NIBS is generally well tolerated, with no significant differences between any intervention and sham/control, and minimal absolute differences across modalities. AEs were predominantly mild and self-limiting, with an AE-related dropout rate of only approximately 1%.

### 4.1 Interpretation of main findings

All NIBS interventions had RRs close to 1.00 compared with sham/control, indicating that NIBS per se does not lead to additional dropout. SUCRA placed iTBS, BL-rTMS, and Dual-tDCS in the top three positions. The favorable ranking of iTBS may relate to its shorter treatment duration (typically 3–8 min per session, versus 20–40 min for conventional rTMS) (10, 93); however, it is unclear whether this reflects superior tolerability or simply fewer opportunities for dropout owing to reduced treatment burden. These rankings should be interpreted with substantial caution: absolute differences were small, all pairwise 95% CrIs included 1.00, several subtypes were informed by a single comparison, and SUCRA compresses uncertainty into a single ordinal position (illustrated by the HD-tDCS P(Best) of 29.5% despite an 11th-place mean rank). They should therefore be regarded as exploratory and hypothesis-generating rather than definitive (94).

### 4.2 Subgroup analyses and sources of heterogeneity

tDCS demonstrated a higher completion rate (96.8%) than rTMS (93.9%), consistent with its greater portability, simpler operation, and lower subjective discomfort (95, 96). Although the AE rate was numerically higher for tDCS (26.6%) than rTMS (11.1%), this discordance between higher AE rates and higher completion rates warrants cautious interpretation, and several non-exclusive explanations should be considered: (1) differential reporting practices, as tDCS trials may more systematically document mild transient skin sensations that rTMS trials might not record; (2); differences in AE monitoring protocols, with tDCS trials potentially using more structured checklists; and (3) differences in study populations or baseline characteristics. The higher tDCS AE rate may therefore partly reflect more rigorous ascertainment rather than a true difference in safety (97, 98).

Stroke phase was associated with completion rate, with the chronic phase highest (97.1%), followed by the subacute (95.9%) and acute (92.8%) phases, while the mixed phase exhibited the lowest completion rate (89.1%). These findings should be interpreted with caution, however, because the completion rate metric cannot distinguish unfavorable attrition (due to adverse events, burden, or lack of benefit) from favorable early termination (such as recovery-driven discharge), particularly relevant in the acute phase where spontaneous recovery is active (27). Furthermore, the mixed-phase subgroup showed substantial heterogeneity (*I*^2^ = 60.6%), suggesting that this estimate may reflect study-level heterogeneity rather than true patient-level phase effects (99, 100). Future studies should systematically document reasons for non-completion to enable more nuanced interpretation of completion rates across stroke phases.

### 4.3 Safety profile

The intervention-group AE rate was 16.4%, predominantly mild and self-resolving, with skin tingling and headache being the most common (101, 102). The AE-related dropout rate of only 1.1%, comparable to control, indicates that AEs were not a major driver of discontinuation (103, 104). Only one study reported serious adverse events (two seizures in the rTMS group), underscoring the need for continued vigilance regarding seizure risk in rTMS, particularly in patients with a history of epilepsy (9, 105).

### 4.4 Comparison with previous studies

Previous systematic reviews have predominantly focused on NIBS efficacy, with completion and tolerability reported as secondary descriptive findings (106, 107). The present study is the first to employ treatment completion rate as the primary outcome, and to compare it across all major NIBS subtypes within a unified NMA framework (108), while providing the most comprehensive AE synthesis for this population to date (109).

### 4.5 Strengths and limitations

Strengths include a comprehensive six-source search with no restriction on study period, a unified NMA of 14 interventions, and extensive subgroup, meta-regression, GRADE, and sensitivity analyses. Several limitations should be acknowledged. First, the star-shaped network precluded formal consistency testing. Second, four subtypes were informed by a single study, yielding imprecise SUCRA rankings; this instability is illustrated by the P(Best) paradox—HD-tDCS ranked 11th yet had the highest P(Best) (29.5%), whereas top-ranked iTBS had only 11.6%—underscoring that these rankings should not be interpreted as definitive evidence of superiority. Additionally, the aggregation of heterogeneous studies spanning multiple stroke stages within the mixed-phase category may confound phase-specific effect estimates in meta-regression. Third, the overall risk of bias was moderate. Fourth, completion rate as a binary outcome is inherently sensitive to protocol length and intensity: shorter protocols mechanically afford fewer opportunities for attrition, so SUCRA rankings may partly reflect protocol brevity rather than intrinsic tolerability, and session-level adherence data are needed in future trials. Fifth, the completion-rate construct cannot distinguish unfavorable dropout from favorable recovery-driven discontinuation. None of the included studies systematically captured the latter, limiting the interpretability of completion rate as a tolerability indicator. Moreover, SUCRA itself compresses heterogeneous evidence into a single ordinal scale, which may obscure meaningful uncertainty across interventions. Sixth, AE ascertainment varied across studies (structured checklists versus spontaneous reporting), contributing to the high AE heterogeneity (I^2^ = 94.2%) and limiting the comparability of AE rates across stimulation types. Finally, only English-language publications were included, and most evidence was of low to moderate GRADE quality.

## 5 Conclusion

This systematic review and network meta-analysis of 55 RCTs demonstrates that NIBS for post-stroke upper limb rehabilitation has favorable tolerability and safety, with an overall completion rate of approximately 95% and no significant differences between any intervention and sham/control; AEs were predominantly mild, with very low AE-related dropout. Exploratory SUCRA rankings placed iTBS, BL-rTMS, and Dual-tDCS in the top three positions; however, these rankings should be considered hypothesis-generating rather than definitive, given that four interventions were informed by only a single study, absolute differences were minimal, and all pairwise credible intervals included 1.00. tDCS showed a slightly higher completion rate than rTMS, and stroke phase was associated with completion rate, with the chronic phase highest (97.1%), followed by subacute (95.9%) and acute (92.8%). Studies with mixed-phase patient populations showed the lowest completion rate (89.1%), though this finding may reflect study-level heterogeneity rather than a true patient-level effect. Nevertheless, these phase-specific differences in completion rates suggest that stroke phase may be an important factor to consider when designing adherence strategies, though future research should prospectively evaluate whether tailored interventions—such as intensified monitoring or psychosocial support for acute-phase patients—can improve treatment completion and clinical outcomes.

## Data Availability

The original contributions presented in the study are included in the article/Supplementary material, further inquiries can be directed to the corresponding author/s.
