# Supplementary material for: Completion rate and safety of non-invasive brain stimulation for upper limb dysfunction after stroke: a systematic review and network meta-analysis
Source: Front Neurol. 2026 Sep 9;17:1853609. doi: 10.3389/fneur.2026.1853609 (PMC13597372; doi:10.3389/fneur.2026.1853609)
Supplement: Supplementary file 1 [file Table_1.DOCX]

**Supplementary Table S1. Sources searched, coverage, and PubMed search strategy**

| **Source** | **Coverage** | **Records (n)** |
| --- | --- | --- |
| PubMed | 1946 to present | 493 |
| Embase | 1947 to present | 566 |
| Cochrane CENTRAL | 1898 to present | 1,733 |
| CINAHL | 1937 to present | 7 |
| WHO ICTRP (registry) | 2007 to present | 409 |
| ClinicalTrials.gov (registry) | 2000 to present | 258 |

***Note:*** *Initial search conducted on January 20, 2025. An updated search using the identical strategy was conducted from January 21, 2025 to April 15, 2026 prior to submission; it identified 47 additional records, none of which met the inclusion criteria.*

| **Set** | **Search terms** |
| --- | --- |
| #1 Stroke | stroke[MeSH] OR stroke OR strokes OR cerebrovascular accident OR cerebral stroke OR apoplexy OR CVA OR acute stroke OR acute cerebrovascular accident [Title/Abstract] (with related entry terms) |
| #2 Upper extremity | upper extremity[MeSH] OR upper limb OR upper extremities OR upper limbs [Title/Abstract] (with related entry terms) |
| #3 NIBS intervention | Transcranial Direct Current Stimulation[MeSH] OR tDCS OR transcranial direct current stimulation OR anodal/cathodal/dual/bilateral/HD-tDCS OR Transcranial Magnetic Stimulation[MeSH] OR rTMS OR TMS OR repetitive transcranial magnetic stimulation OR high-/low-frequency rTMS OR bilateral rTMS OR theta burst stimulation OR TBS OR iTBS OR cTBS [Title/Abstract] (with related entry terms) |
| #4 RCT | randomized controlled trial[Publication Type] OR randomized/randomised controlled trial OR RCT OR (randomized AND controlled) NOT (case report OR case series OR review[Publication Type] OR meta-analysis[Publication Type]) |
| Final | #1 AND #2 AND #3 AND #4 |

***Note:*** *The strategy was adapted for Embase, Cochrane CENTRAL, CINAHL, WHO ICTRP, and ClinicalTrials.gov with appropriate syntax modifications.*

**Supplementary Table S2. Full study-level dataset**

| **ID** | **First author (Year)** | **Country** | **Phase** | **Subtype** | **Comparator** | **Int. enrolled** | **Int. completed** | **Int. CR%** | **Con. enrolled** | **Con. completed** | **Con. CR%** | **Sessions** | **Setting** | **Stim. site** |
| --- | --- | --- | --- | --- | --- | --- | --- | --- | --- | --- | --- | --- | --- | --- |
| 1 | Harvey (2018) | USA | Subacute-chronic | LF-rTMS | Sham | 132 | 124 | 93.9 | 67 | 63 | 94.0 | 18 | Outpatient | Contralesional M1 |
| 2 | Kim (2020) | South Korea | Subacute | LF-rTMS | Sham | 40 | 36 | 90.0 | 37 | 37 | 100.0 | 10 | Hospital | Contralesional M1 |
| 3 | Chang (2010) | South Korea | Subacute | HF-rTMS | Sham | 18 | 18 | 100.0 | 10 | 10 | 100.0 | 10 | Inpatient | Ipsilesional M1 |
| 4 | Viana (2014) | Brazil | Chronic | A-tDCS | Sham | 10 | 10 | 100.0 | 10 | 10 | 100.0 | 15 | Outpatient | Ipsilesional M1 |
| 5 | Wang (2020) | China | Subacute | HF-rTMS | Sham | 15 | 15 | 100.0 | 15 | 15 | 100.0 | 14 | Inpatient | Contralesional M1 |
| 5 | Wang (2020) | China | Subacute | LF-rTMS | Sham | 15 | 15 | 100.0 | 15 | 15 | 100.0 | 14 | Inpatient | Contralesional M1 |
| 6 | Long (2018) | China | Acute | LF-rTMS | Sham | 21 | 21 | 100.0 | 20 | 20 | 100.0 | 15 | Inpatient | Contralesional M1 |
| 6 | Long (2018) | China | Acute | BL-rTMS | Sham | 21 | 21 | 100.0 | 20 | 20 | 100.0 | 15 | Inpatient | Bilateral M1 |
| 7 | Hsu (2023) | Taiwan | Subacute | Dual-tDCS | Sham | 13 | 13 | 100.0 | 14 | 14 | 100.0 | 20 | Inpatient | Bilateral M1 |
| 8 | Ilić (2016) | Serbia | Chronic | A-tDCS | Sham | 14 | 14 | 100.0 | 12 | 11 | 91.7 | 10 | Outpatient | Ipsilesional M1 |
| 9 | Guan (2017) | China | Acute | HF-rTMS | Sham | 21 | 13 | 61.9 | 21 | 14 | 66.7 | 10 | Inpatient | Ipsilesional M1 |

| **Supplementary Table S2. (Continued)** | | | | | | | | | | | | | | |
| --- | --- | --- | --- | --- | --- | --- | --- | --- | --- | --- | --- | --- | --- | --- |
| **ID** | **First author (Year)** | **Country** | **Phase** | **Subtype** | **Comparator** | **Int. enrolled** | **Int. completed** | **Int. CR%** | **Con. enrolled** | **Con. completed** | **Con. CR%** | **Sessions** | **Setting** | **Stim. site** |
| 10 | Lüdemann-Podubecká (2015) | Germany | Subacute | LF-rTMS | Sham | 20 | 20 | 100.0 | 20 | 20 | 100.0 | 15 | Inpatient | Contralesional M1 |
| 11 | Tedesco Triccas (2015) | UK | Mixed | A-tDCS | Sham | 12 | 11 | 91.7 | 11 | 11 | 100.0 | 18 | Outpatient | Ipsilesional M1 |
| 12 | Wang (2014) | Taiwan | Subacute | Combined | Sham | 17 | 17 | 100.0 | 16 | 16 | 100.0 | 20 | Inpatient | Bilateral (contra-then-ipsi) |
| 12 | Wang (2014) | Taiwan | Subacute | Combined | Sham | 15 | 15 | 100.0 | 16 | 16 | 100.0 | 20 | Inpatient | Bilateral (ipsi-then-contra) |
| 13 | Hosomi (2016) | Japan | Subacute | HF-rTMS | Sham | 20 | 18 | 90.0 | 21 | 21 | 100.0 | 10 | Inpatient | Ipsilesional M1 |
| 14 | Khan (2019) | India | Acute | BL-TBS | Standard care | 20 | 20 | 100.0 | 20 | 20 | 100.0 | 12 | Inpatient/Outpatient | Bilateral M1 |
| 15 | Hesse (2011) | Germany | Subacute | A-tDCS | Sham | 32 | 31 | 96.9 | 32 | 32 | 100.0 | 30 | Inpatient | Ipsilesional M1 |
| 15 | Hesse (2011) | Germany | Subacute | C-tDCS | Sham | 32 | 31 | 96.9 | 32 | 32 | 100.0 | 30 | Inpatient | Contralesional M1 |
| 16 | Unger (2023) | USA | Chronic | A-tDCS | Sham | 11 | 9 | 81.8 | 14 | 11 | 78.6 | 30 | Outpatient | Ipsilesional PMC |
| 17 | Youssef (2023) | Egypt | Subacute | BL-tDCS | Sham | 11 | 11 | 100.0 | 11 | 11 | 100.0 | 12 | Outpatient | Bilateral M1 |

| **Supplementary Table S2. (Continued)** | | | | | | | | | | | | | | |
| --- | --- | --- | --- | --- | --- | --- | --- | --- | --- | --- | --- | --- | --- | --- |
| **ID** | **First author (Year)** | **Country** | **Phase** | **Subtype** | **Comparator** | **Int. enrolled** | **Int. completed** | **Int. CR%** | **Con. enrolled** | **Con. completed** | **Con. CR%** | **Sessions** | **Setting** | **Stim. site** |
| 17 | Youssef (2023) | Egypt | Subacute | A-tDCS | Sham | 13 | 13 | 100.0 | 11 | 11 | 100.0 | 12 | Outpatient | Ipsilesional M1 |
| 18 | Zhang (2022) | China | Chronic | Priming iTBS | Sham | 14 | 14 | 100.0 | 14 | 14 | 100.0 | 10 | Laboratory | Ipsilesional M1 |
| 18 | Zhang (2022) | China | Chronic | Non-priming iTBS | Sham | 14 | 14 | 100.0 | 14 | 14 | 100.0 | 10 | Laboratory | Ipsilesional M1 |
| 19 | Conforto (2012) | Brazil | Subacute | LF-rTMS | Sham | 15 | 15 | 100.0 | 15 | 14 | 93.3 | 10 | Outpatient | Contralesional M1 |
| 20 | Chervyakov (2018) | Russia | Subacute-chronic | LF-rTMS | Sham | 18 | 11 | 61.1 | 11 | 10 | 90.9 | 10 | Inpatient | Contralesional M1 |
| 20 | Chervyakov (2018) | Russia | Subacute-chronic | HF-rTMS | Sham | 18 | 13 | 72.2 | 11 | 10 | 90.9 | 10 | Inpatient | Ipsilesional M1 |
| 21 | Chen (2021) | China | Acute | BL-rTMS | Sham | 32 | 31 | 96.9 | 32 | 30 | 93.8 | 20 | Inpatient | Bilateral M1 |
| 21 | Chen (2021) | China | Acute | HF-rTMS | Sham | 32 | 25 | 78.1 | 32 | 25 | 78.1 | 20 | Inpatient | Ipsilesional M1 |
| 21 | Chen (2021) | China | Acute | LF-rTMS | Sham | 32 | 25 | 78.1 | 32 | 25 | 78.1 | 20 | Inpatient | Contralesional M1 |
| 22 | Bornheim (2019) | Belgium | Acute | A-tDCS | Sham | 25 | 25 | 100.0 | 25 | 25 | 100.0 | 20 | Inpatient | Ipsilesional M1 |
| 23 | Bolognini (2020) | Italy | Acute | Dual-tDCS | Sham | 16 | 16 | 100.0 | 16 | 16 | 100.0 | 10 | Inpatient | Bilateral M1 |

**Supplementary Table S2. (Continued)**

| **ID** | **First author (Year)** | **Country** | **Phase** | **Subtype** | **Comparator** | **Int. enrolled** | **Int. completed** | **Int. CR%** | **Con. enrolled** | **Con. completed** | **Con. CR%** | **Sessions** | **Setting** | **Stim. site** |
| --- | --- | --- | --- | --- | --- | --- | --- | --- | --- | --- | --- | --- | --- | --- |
| 24 | Straudi (2016) | Italy | Mixed | Dual-tDCS | Sham | 12 | 12 | 100.0 | 11 | 11 | 100.0 | 10 | NR | Bilateral M1 |
| 25 | Seniów (2012) | Poland | Subacute | LF-rTMS | Sham | 20 | 20 | 100.0 | 20 | 20 | 100.0 | 15 | Inpatient | Contralesional M1 |
| 26 | Mazzoleni (2019) | Italy | Subacute | A-tDCS | Sham | 20 | 20 | 100.0 | 20 | 19 | 95.0 | 30 | Inpatient | Ipsilesional M1 |
| 27 | Etoh (2013) | Japan | Chronic | LF-rTMS | Sham | 18 | 18 | 100.0 | 18 | 18 | 100.0 | 10 | NR | Contralesional M1 |
| 28 | Emara (2010) | Egypt | Chronic | HF-rTMS | Sham | 20 | 20 | 100.0 | 20 | 20 | 100.0 | 10 | NR | Ipsilesional M1 |
| 28 | Emara (2010) | Egypt | Chronic | LF-rTMS | Sham | 20 | 20 | 100.0 | 20 | 20 | 100.0 | 10 | NR | Contralesional M1 |
| 29 | Shim (2023) | South Korea | Subacute | HF-rTMS | Sham | 17 | 14 | 82.4 | 16 | 16 | 100.0 | 12 | Inpatient | Ipsilesional M1 |
| 30 | Di Lazzaro (2016) | Italy | Chronic | cTBS | Sham | 10 | 8 | 80.0 | 10 | 9 | 90.0 | 10 | Outpatient | Ipsilesional M1 |
| 31 | Lai (2015) | Taiwan | Chronic | iTBS | Sham | 21 | 21 | 100.0 | 17 | 17 | 100.0 | 10 | Outpatient | Ipsilesional M1 |
| 32 | Liao (2025) | Taiwan | Chronic | A-tDCS | Sham | 12 | 12 | 100.0 | 12 | 12 | 100.0 | 20 | Hospital | Ipsilesional PMC |
| 32 | Liao (2025) | Taiwan | Chronic | A-tDCS | Sham | 12 | 12 | 100.0 | 12 | 12 | 100.0 | 20 | Hospital | Ipsilesional M1 |
| 33 | Rose (2014) | USA | Chronic | LF-rTMS | Sham | 11 | 9 | 81.8 | 10 | 10 | 100.0 | 16 | Outpatient | Contralesional M1 |

| **Supplementary Table S2. (Continued)** | | | | | | | | | | | | | | |
| --- | --- | --- | --- | --- | --- | --- | --- | --- | --- | --- | --- | --- | --- | --- |
| **ID** | **First author (Year)** | **Country** | **Phase** | **Subtype** | **Comparator** | **Int. enrolled** | **Int. completed** | **Int. CR%** | **Con. enrolled** | **Con. completed** | **Con. CR%** | **Sessions** | **Setting** | **Stim. site** |
| 34 | Moslemi Haghighi (2021) | Iran | Subacute | HF-rTMS | Standard care | 10 | 10 | 100.0 | 10 | 10 | 100.0 | 10 | NR | Ipsilesional M1 |
| 35 | Ke (2020) | China | Acute | HF-rTMS | Sham | 16 | 14 | 87.5 | 16 | 15 | 93.8 | 10 | Inpatient | Ipsilesional M1 |
| 35 | Ke (2020) | China | Acute | HF-rTMS | Sham | 16 | 15 | 93.8 | 16 | 15 | 93.8 | 10 | Inpatient | Ipsilesional M1 |
| 36 | Shaheiwola (2018) | China | Chronic | BL-tDCS | Sham | 15 | 15 | 100.0 | 15 | 15 | 100.0 | 20 | Hospital | Bilateral M1 |
| 37 | Ni (2023) | China | Subacute | HF-rTMS | Sham | 18 | 18 | 100.0 | 18 | 15 | 83.3 | 20 | Inpatient | Ipsilesional M1 |
| 38 | Chen (2025) | China | Subacute | iTBS | Sham | 25 | 25 | 100.0 | 25 | 23 | 92.0 | 10 | Inpatient | Ipsilesional M1 |
| 39 | Wang (2023) | China | Chronic | LF-rTMS | Standard care | 23 | 23 | 100.0 | 23 | 23 | 100.0 | 14 | Inpatient | Contralesional M1 |
| 40 | Wu (2023) | China | Subacute | HF-rTMS | Sham | 15 | 15 | 100.0 | 15 | 15 | 100.0 | 15 | Inpatient | Ipsilesional M1 |
| 41 | Yang (2021) | China | Subacute | HF-rTMS | Sham | 14 | 12 | 85.7 | 15 | 13 | 86.7 | 10 | Inpatient/Outpatient | Ipsilesional M1 |
| 42 | Edwards (2018) | USA | Chronic | A-tDCS | Sham | 41 | 40 | 97.6 | 41 | 37 | 90.2 | 36 | Outpatient | Ipsilesional M1 |
| 43 | Palimeris (2022) | Canada | Chronic | A-tDCS | Sham | 48 | 48 | 100.0 | 42 | 42 | 100.0 | 12 | Outpatient | Ipsilesional M1 |

**Supplementary Table S2. (Continued)**

| **ID** | **First author (Year)** | **Country** | **Phase** | **Subtype** | **Comparator** | **Int. enrolled** | **Int. completed** | **Int. CR%** | **Con. enrolled** | **Con. completed** | **Con. CR%** | **Sessions** | **Setting** | **Stim. site** |
| --- | --- | --- | --- | --- | --- | --- | --- | --- | --- | --- | --- | --- | --- | --- |
| 44 | Liu (2025) | China | Subacute | LF-rTMS | Standard care | 35 | 34 | 97.1 | 35 | 30 | 85.7 | 40 | Inpatient | Contralesional M1 |
| 45 | Chen (2021) | China | Acute | BL-rTMS | Sham | 24 | 22 | 91.7 | 24 | 22 | 91.7 | 20 | Inpatient | Bilateral M1 |
| 46 | Gao (2025) | China | Subacute | Dual-tDCS | Sham | 22 | 20 | 90.9 | 23 | 19 | 82.6 | 10 | Inpatient | Bilateral M1 |
| 47 | Tang (2024) | China | Mixed | iTBS | Sham | 15 | 15 | 100.0 | 15 | 14 | 93.3 | 15 | Inpatient | Ipsilesional M1 |
| 47 | Tang (2024) | China | Mixed | cTBS | Sham | 15 | 14 | 93.3 | 15 | 14 | 93.3 | 15 | Inpatient | Contralesional M1 |
| 48 | Morone (2022) | Italy | Chronic | Dual-tDCS | Sham | 33 | 33 | 100.0 | 33 | 33 | 100.0 | 10 | Outpatient | Bilateral M1 |
| 49 | Lee SH (2025) | South Korea | Chronic | HD-tDCS | Sham | 14 | 12 | 85.7 | 14 | 12 | 85.7 | 10 | Outpatient | Bilateral M1 |
| 50 | Chen (2019) | Taiwan | Chronic | iTBS | Sham | 12 | 11 | 91.7 | 11 | 11 | 100.0 | 10 | Inpatient | Ipsilesional M1 |
| 51 | Salazar (2020) | Brazil | Chronic | Dual-tDCS | Sham | 15 | 15 | 100.0 | 15 | 15 | 100.0 | 10 | Outpatient | Bilateral M1 |
| 52 | Pinto (2021) | India | Mixed | Dual-tDCS | Sham | 31 | 29 | 93.5 | 29 | 24 | 82.8 | 24 | Inpatient | Bilateral M1 |
| 53 | Zheng (2015) | China | Subacute | LF-rTMS | Sham | 58 | 55 | 94.8 | 54 | 53 | 98.1 | 24 | Inpatient | Contralesional M1 |

**Supplementary Table S2. (Continued)**

| **ID** | **First author (Year)** | **Country** | **Phase** | **Subtype** | **Comparator** | **Int. enrolled** | **Int. completed** | **Int. CR%** | **Con. enrolled** | **Con. completed** | **Con. CR%** | **Sessions** | **Setting** | **Stim. site** |
| --- | --- | --- | --- | --- | --- | --- | --- | --- | --- | --- | --- | --- | --- | --- |
| 54 | Zhao (2022) | China | Acute | Dual-tDCS | Standard care | 30 | 30 | 100.0 | 30 | 30 | 100.0 | 12 | Inpatient | Bilateral M1 |
| 55 | Yao (2020) | China | Subacute-chronic | C-tDCS | Sham | 22 | 20 | 90.9 | 20 | 20 | 100.0 | 10 | Inpatient | Contralesional M1 |

*Note: One row per comparison (k = 68). CR% = completion rate; Int. = intervention arm; Con. = control arm; Stim. site = stimulation site; NR = not reported.*

**Supplementary Table S3. Reasons for dropout**

| **ID** | **Group** | **AE-related withdrawal** | **Adherence/personal reasons** | **Disease-related** | **Lost to follow-up** | **Other/Unspecified** | **Total dropout** |
| --- | --- | --- | --- | --- | --- | --- | --- |
| 1 | Intervention | 4 | 1 | 0 | 0 | 3 | 8 |
| 1 | Control | 1 | 2 | 0 | 0 | 1 | 4 |
| 2 | Intervention | 0 | 4 | 0 | 0 | 0 | 4 |
| 2 | Control | 0 | 0 | 0 | 0 | 0 | 0 |
| 8 | Intervention | 0 | 0 | 0 | 0 | 0 | 0 |
| 8 | Control | 0 | 0 | 1 | 0 | 0 | 1 |
| 9 | Intervention | 0 | 0 | 0 | 8 | 0 | 8 |
| 9 | Control | 0 | 0 | 0 | 7 | 0 | 7 |
| 11 | Intervention | 1 | 0 | 0 | 0 | 0 | 1 |
| 11 | Control | 0 | 0 | 0 | 0 | 0 | 0 |
| 13 | Intervention | 1 | 1 | 0 | 0 | 0 | 2 |
| 13 | Control | 0 | 0 | 0 | 0 | 0 | 0 |
| 15 | Intervention(A-tDCS) | 0 | 0 | 0 | 0 | 1 | 1 |
| 15 | Intervention(C-tDCS) | 0 | 0 | 1 | 0 | 0 | 1 |
| 15 | Control | 0 | 0 | 0 | 0 | 0 | 0 |
| 16 | Intervention | 0 | 0 | 0 | 2 | 0 | 2 |
| 16 | Control | 0 | 0 | 0 | 3 | 0 | 3 |
| 19 | Intervention | 0 | 0 | 0 | 0 | 0 | 0 |
| 19 | Control | 0 | 0 | 1 | 0 | 0 | 1 |
| 20 | Intervention(rTMS pooled) | 0 | 0 | 0 | 0 | 0 | 21 |
| 20 | Control | 0 | 0 | 0 | 0 | 1 | 1 |
| 21 | Intervention(BL) | 0 | 0 | 0 | 6 | 1 | 7 |
| 21 | Control(BLshared) | 0 | 0 | 0 | 5 | 2 | 7 |
| 21 | Intervention(HF) | 0 | 0 | 0 | 5 | 2 | 7 |
| 21 | Control(HFshared) | 0 | 0 | 0 | 5 | 2 | 7 |

**Supplementary Table S3. (Continued)**

| **ID** | **Group** | **AE-related withdrawal** | **Adherence/personal reasons** | **Disease-related** | **Lost to follow-up** | **Other/Unspecified** | **Total dropout** |
| --- | --- | --- | --- | --- | --- | --- | --- |
| 21 | Intervention(LF) | 0 | 0 | 0 | 7 | 0 | 7 |
| 21 | Control(LFshared) | 0 | 0 | 0 | 5 | 2 | 7 |
| 26 | Intervention | 0 | 0 | 0 | 0 | 0 | 0 |
| 26 | Control | 0 | 0 | 0 | 0 | 1 | 1 |
| 29 | Intervention | 0 | 1 | 0 | 2 | 0 | 3 |
| 29 | Control | 0 | 0 | 0 | 0 | 0 | 0 |
| 30 | Intervention | 0 | 1 | 0 | 0 | 1 | 2 |
| 30 | Control | 0 | 1 | 0 | 0 | 0 | 1 |
| 33 | Intervention | 0 | 1 | 0 | 1 | 0 | 2 |
| 33 | Control | 0 | 0 | 0 | 0 | 0 | 0 |
| 35 | Intervention(Short ITI) | 0 | 0 | 0 | 1 | 2 | 3 |
| 35 | Control(Short ITIshared) | 0 | 0 | 0 | 2 | 1 | 3 |
| 35 | Intervention(Long ITI) | 0 | 0 | 0 | 1 | 1 | 2 |
| 35 | Control(Long ITIshared) | 0 | 0 | 0 | 2 | 1 | 3 |
| 37 | Intervention | 0 | 0 | 0 | 0 | 0 | 0 |
| 37 | Control | 0 | 0 | 3 | 0 | 0 | 3 |
| 38 | Intervention | 0 | 0 | 0 | 0 | 0 | 0 |
| 38 | Control | 0 | 1 | 0 | 1 | 0 | 2 |
| 41 | Intervention | 0 | 0 | 0 | 0 | 2 | 2 |
| 41 | Control | 0 | 0 | 0 | 0 | 2 | 2 |
| 42 | Intervention | 0 | 0 | 1 | 0 | 0 | 1 |
| 42 | Control | 0 | 0 | 4 | 0 | 0 | 4 |
| 44 | Intervention | 0 | 0 | 0 | 0 | 1 | 1 |
| 44 | Control | 0 | 0 | 0 | 0 | 5 | 5 |
| 45 | Intervention | 0 | 0 | 0 | 1 | 1 | 2 |
| 45 | Control | 0 | 0 | 1 | 1 | 0 | 2 |

**Supplementary Table S3. (Continued)**

| **ID** | **Group** | **AE-related withdrawal** | **Adherence/personal reasons** | **Disease-related** | **Lost to follow-up** | **Other/Unspecified** | **Total dropout** |
| --- | --- | --- | --- | --- | --- | --- | --- |
| 46 | Intervention | 0 | 0 | 0 | 0 | 2 | 2 |
| 46 | Control | 0 | 0 | 0 | 0 | 4 | 4 |
| 47 | Intervention(iTBS) | 0 | 0 | 0 | 0 | 0 | 0 |
| 47 | Intervention(cTBS) | 0 | 1 | 0 | 0 | 0 | 1 |
| 47 | Control | 0 | 0 | 0 | 0 | 1 | 1 |
| 49 | Intervention | 0 | 1 | 0 | 0 | 1 | 2 |
| 49 | Control | 0 | 1 | 0 | 0 | 1 | 2 |
| 50 | Intervention | 0 | 0 | 0 | 1 | 0 | 1 |
| 50 | Control | 0 | 0 | 0 | 0 | 0 | 0 |
| 52 | Intervention | 1 | 0 | 0 | 0 | 1 | 2 |
| 52 | Control | 0 | 3 | 1 | 0 | 1 | 5 |
| 53 | Intervention | 0 | 3 | 0 | 0 | 0 | 3 |
| 53 | Control | 0 | 1 | 0 | 0 | 0 | 1 |
| 55 | Intervention | 0 | 1 | 1 | 0 | 0 | 2 |
| 55 | Control | 0 | 0 | 0 | 0 | 0 | 0 |

*Note: Only studies with reported dropout reasons are included. Studies not listed had 100% completion or did not report dropout reasons. AE = adverse event.*

**Supplementary Table S4. Risk-of-bias judgments (RoB 2), per study and domain**

| **ID** | **Study** | **D1 Randomization** | **D2 Deviations** | **D3 Missing data** | **D4 Measurement** | **D5 Selection** | **Overall** |
| --- | --- | --- | --- | --- | --- | --- | --- |
| 1 | Harvey 2018 | Low | Low | Low | Low | Low | Low |
| 2 | Kim 2020 | Low | Low | Low | Low | Low | Low |
| 3 | Chang 2010 | High | Low | Low | Low | Some concerns | High |
| 4 | Viana 2014 | Some concerns | Low | Low | Low | Some concerns | Some concerns |
| 5 | Wang 2020 | Some concerns | Low | Low | Low | Low | Some concerns |
| 6 | Long 2018 | Some concerns | Low | Low | Low | Some concerns | Some concerns |
| 7 | Hsu 2023 | Low | Low | Low | Low | Low | Low |
| 8 | Ilić 2016 | Some concerns | Low | Low | Low | Some concerns | Some concerns |
| 9 | Guan 2017 | Some concerns | High | High | Low | Some concerns | High |
| 10 | Lüdemann-Podubecká 2015 | Some concerns | Low | Low | Low | High | High |
| 11 | Tedesco Triccas 2015 | Low | Low | Low | Low | Low | Low |
| 12 | Wang 2014 | Low | Low | Low | Low | Some concerns | Some concerns |
| 13 | Hosomi 2016 | Low | Low | Low | Low | Low | Low |
| 14 | Khan 2019 | Some concerns | Low | Low | Low | Some concerns | Some concerns |
| 15 | Hesse 2011 | Low | Low | Low | Low | Low | Low |
| 16 | Unger 2023 | Low | Some concerns | Low | Low | High | High |
| 17 | Youssef 2023 | Some concerns | Low | Low | Low | Low | Some concerns |
| 18 | Zhang 2022 | Low | Low | Low | Low | Low | Low |
| 19 | Conforto 2012 | Low | Low | Low | Low | Some concerns | Some concerns |
| 20 | Chervyakov 2018 | Low | High | High | Low | Some concerns | High |
| 21 | Chen 2021 | Low | Some concerns | Low | Some concerns | Low | Some concerns |
| 22 | Bornheim 2019 | Low | Low | Low | Low | Some concerns | Some concerns |
| 23 | Bolognini 2020 | Low | Low | Low | Low | Some concerns | Some concerns |

**Supplementary Table S4. (Continued)**

| **ID** | **Study** | **D1 Randomization** | **D2 Deviations** | **D3 Missing data** | **D4 Measurement** | **D5 Selection** | **Overall** |
| --- | --- | --- | --- | --- | --- | --- | --- |
| 24 | Straudi 2016 | Some concerns | Low | Low | Low | Some concerns | Some concerns |
| 25 | Seniów 2012 | Low | Low | Low | Low | Some concerns | Some concerns |
| 26 | Mazzoleni 2019 | Some concerns | Low | Low | Low | Low | Some concerns |
| 27 | Etoh 2013 | Some concerns | Low | Low | Low | Some concerns | Some concerns |
| 28 | Emara 2010 | Low | Low | Low | Low | Some concerns | Some concerns |
| 29 | Shim 2023 | Some concerns | Some concerns | Low | Some concerns | Low | Some concerns |
| 30 | Di Lazzaro 2016 | Low | Some concerns | Low | Low | Some concerns | Some concerns |
| 31 | Lai 2015 | Some concerns | Low | Low | Low | Low | Some concerns |
| 32 | Liao 2025 | Low | Low | Low | Low | Low | Low |
| 33 | Rose 2014 | Low | Low | Low | Low | Low | Low |
| 34 | Moslemi Haghighi 2021 | Some concerns | Low | Low | Low | Some concerns | Some concerns |
| 35 | Ke 2020 | Some concerns | Low | Low | Low | Some concerns | Some concerns |
| 36 | Shaheiwola 2018 | Some concerns | Low | Low | Some concerns | High | High |
| 37 | Ni 2023 | Some concerns | Low | Low | High | Some concerns | High |
| 38 | Chen 2025 | Low | Low | Low | Low | Some concerns | Some concerns |
| 39 | Wang 2023 | Low | Low | Low | High | Some concerns | High |
| 40 | Wu 2023 | Some concerns | Low | Low | Low | Low | Some concerns |
| 41 | Yang 2021 | Some concerns | Low | Low | High | Some concerns | High |
| 42 | Edwards 2018 | Some concerns | Low | Low | Low | Low | Some concerns |
| 43 | Palimeris 2022 | Some concerns | Low | Low | Low | Low | Some concerns |
| 44 | Liu 2025 | Low | Low | Low | Low | Low | Low |
| 45 | Chen 2021b | Low | Low | Low | Low | Low | Low |
| 46 | Gao 2025 | Low | Low | Low | Low | Low | Low |
| 47 | Tang 2024 | Low | Low | Low | Low | Some concerns | Some concerns |

**Supplementary Table S4. (Continued)**

| **ID** | **Study** | **D1 Randomization** | **D2 Deviations** | **D3 Missing data** | **D4 Measurement** | **D5 Selection** | **Overall** |
| --- | --- | --- | --- | --- | --- | --- | --- |
| 48 | Morone 2022 | Low | Low | Low | Low | Low | Low |
| 49 | Lee SH 2025 | High | Low | Low | Low | Low | High |
| 50 | Chen 2019 | Some concerns | Some concerns | Low | Low | Some concerns | Some concerns |
| 51 | Salazar 2020 | Low | Low | Low | Low | Low | Low |
| 52 | Pinto 2021 | Low | Low | Low | Low | Low | Low |
| 53 | Zheng 2015 | Some concerns | Some concerns | Low | Low | Some concerns | Some concerns |
| 54 | Zhao 2022 | Some concerns | Low | Low | Some concerns | Some concerns | Some concerns |
| 55 | Yao 2020 | Low | Some concerns | Low | Low | Some concerns | Some concerns |

*Note: D1 = Randomization process; D2 = Deviations from intended interventions; D3 = Missing outcome data; D4 = Measurement of the outcome; D5 = Selection of the reported result. Low = low risk of bias; High = high risk of bias; Some concerns = some concerns about bias.*

**Supplementary Table S5. Heterogeneity statistics by subgroup**

| **Subgroup** | **Level** | **k** | **I2** | **Q** | **Q_p** | **tau2** |
| --- | --- | --- | --- | --- | --- | --- |
| Intervention Subtype | A-tDCS | 12 | 0.00 | 8.83 | 0.64 | 0.00 |
| Intervention Subtype | BL-TBS | 1 | 0.00 | 0.00 | 1.00 | 0.00 |
| Intervention Subtype | BL-rTMS | 3 | 3.90 | 2.08 | 0.35 | 0.00 |
| Intervention Subtype | BL-tDCS | 2 | 0.00 | 0.01 | 0.92 | 0.00 |
| Intervention Subtype | C-tDCS | 2 | 0.00 | 0.80 | 0.37 | 0.00 |
| Intervention Subtype | Combined | 2 | 0.00 | 0.00 | 0.97 | 0.00 |
| Intervention Subtype | Dual-tDCS | 8 | 0.00 | 6.10 | 0.53 | 0.00 |
| Intervention Subtype | HD-tDCS | 1 | 0.00 | 0.00 | 1.00 | 0.00 |
| Intervention Subtype | HF-rTMS | 14 | 64.80 | 36.90 | 0.00 | 0.10 |
| Intervention Subtype | LF-rTMS | 15 | 62.40 | 37.22 | 0.00 | 0.05 |
| Intervention Subtype | Non-priming iTBS | 1 | 0.00 | 0.00 | 1.00 | 0.00 |
| Intervention Subtype | Priming iTBS | 1 | 0.00 | 0.00 | 1.00 | 0.00 |
| Intervention Subtype | cTBS | 2 | 0.00 | 0.88 | 0.35 | 0.00 |
| Intervention Subtype | iTBS | 4 | 0.00 | 2.30 | 0.51 | 0.00 |
| Stroke Phase | Acute | 13 | 72.60 | 43.81 | 0.00 | 0.11 |
| Stroke Phase | Chronic | 21 | 12.80 | 22.93 | 0.29 | 0.01 |
| Stroke Phase | Mixed | 9 | 60.60 | 20.30 | 0.01 | 0.06 |
| Stroke Phase | Subacute | 25 | 0.00 | 21.30 | 0.62 | 0.00 |
| Stimulation Type | rTMS | 43 | 56.80 | 97.27 | 0.00 | 0.06 |

**Supplementary Table S5. (Continued)**

| **Subgroup** | **Level** | **k** | **I2** | **Q** | **Q_p** | **tau2** |
| --- | --- | --- | --- | --- | --- | --- |
| Stimulation Type | tDCS | 25 | 0.00 | 21.18 | 0.63 | 0.00 |
| Treatment Setting | Inpatient | 41 | 56.80 | 92.61 | 0.00 | 0.06 |
| Treatment Setting | Laboratory | 2 | 0.00 | 0.00 | 1.00 | 0.00 |
| Treatment Setting | Other/NR | 9 | 0.00 | 5.55 | 0.70 | 0.00 |
| Treatment Setting | Outpatient | 16 | 34.30 | 22.84 | 0.09 | 0.02 |

*Note: k = number of comparisons; I² = heterogeneity statistic; Q = Cochran's Q statistic; Q_p = P-value for Q test; tau² = between-study variance.*

**Supplementary Table S6. Leave-one-out sensitivity analysis**

| **Excluded_Study** | **Pooled_Rate** | **CI_Lower** | **CI_Upper** | **I2** | **k** |
| --- | --- | --- | --- | --- | --- |
| None (Overall) | 94.94 | 93.26 | 96.38 | 46.80 | 68 |
| Harvey 2018 | 94.97 | 93.24 | 96.46 | 47.40 | 67 |
| Kim 2020 | 95.04 | 93.36 | 96.48 | 46.70 | 67 |
| Chang 2010 | 94.87 | 93.17 | 96.34 | 47.20 | 67 |
| Viana 2014 | 94.90 | 93.21 | 96.37 | 47.50 | 67 |
| Wang 2020 | 94.88 | 93.18 | 96.35 | 47.30 | 67 |
| Wang 2020 | 94.88 | 93.18 | 96.35 | 47.30 | 67 |
| Long 2018 | 94.85 | 93.15 | 96.33 | 47.00 | 67 |
| Long 2018 | 94.85 | 93.15 | 96.33 | 47.00 | 67 |
| Hsu 2023 | 94.89 | 93.19 | 96.35 | 47.40 | 67 |
| Ilić 2016 | 94.88 | 93.19 | 96.35 | 47.30 | 67 |
| Guan 2017 | 95.23 | 93.70 | 96.56 | 39.40 | 67 |
| Lüdemann-Podubecká 2015 | 94.86 | 93.16 | 96.33 | 47.10 | 67 |
| Tedesco Triccas 2015 | 94.99 | 93.31 | 96.44 | 47.30 | 67 |
| Wang 2014 | 94.87 | 93.17 | 96.34 | 47.20 | 67 |
| Wang 2014 | 94.88 | 93.18 | 96.35 | 47.30 | 67 |
| Hosomi 2016 | 95.02 | 93.34 | 96.46 | 47.10 | 67 |
| Khan 2019 | 94.86 | 93.16 | 96.33 | 47.10 | 67 |
| Hesse 2011 | 94.92 | 93.22 | 96.40 | 47.60 | 67 |
| Hesse 2011 | 94.92 | 93.22 | 96.40 | 47.60 | 67 |
| Unger 2023 | 95.05 | 93.39 | 96.48 | 46.40 | 67 |
| Youssef 2023 | 94.90 | 93.20 | 96.36 | 47.40 | 67 |
| Youssef 2023 | 94.89 | 93.19 | 96.35 | 47.40 | 67 |
| Zhang 2022 | 94.88 | 93.19 | 96.35 | 47.30 | 67 |
| Zhang 2022 | 94.88 | 93.19 | 96.35 | 47.30 | 67 |
| Conforto 2012 | 94.88 | 93.18 | 96.35 | 47.30 | 67 |

**Supplementary Table S6. (Continued)**

| **Excluded_Study** | **Pooled_Rate** | **CI_Lower** | **CI_Upper** | **I2** | **k** |
| --- | --- | --- | --- | --- | --- |
| Chervyakov 2018 | 95.21 | 93.67 | 96.55 | 40.40 | 67 |
| Chervyakov 2018 | 95.15 | 93.54 | 96.53 | 43.70 | 67 |
| Chen 2021 | 94.92 | 93.22 | 96.40 | 47.60 | 67 |
| Chen 2021 | 95.16 | 93.56 | 96.55 | 43.30 | 67 |
| Chen 2021 | 95.16 | 93.56 | 96.55 | 43.30 | 67 |
| Bornheim 2019 | 94.84 | 93.14 | 96.31 | 46.80 | 67 |
| Bolognini 2020 | 94.88 | 93.18 | 96.34 | 47.30 | 67 |
| Straudi 2016 | 94.89 | 93.20 | 96.36 | 47.40 | 67 |
| Seniów 2012 | 94.86 | 93.16 | 96.33 | 47.10 | 67 |
| Mazzoleni 2019 | 94.86 | 93.16 | 96.33 | 47.10 | 67 |
| Etoh 2013 | 94.87 | 93.17 | 96.34 | 47.20 | 67 |
| Emara 2010 | 94.86 | 93.16 | 96.33 | 47.10 | 67 |
| Emara 2010 | 94.86 | 93.16 | 96.33 | 47.10 | 67 |
| Shim 2023 | 95.07 | 93.42 | 96.50 | 46.00 | 67 |
| Di Lazzaro 2016 | 95.05 | 93.40 | 96.48 | 46.30 | 67 |
| Lai 2015 | 94.85 | 93.15 | 96.33 | 47.00 | 67 |
| Liao 2025 | 94.89 | 93.20 | 96.36 | 47.40 | 67 |
| Liao 2025 | 94.89 | 93.20 | 96.36 | 47.40 | 67 |
| Rose 2014 | 95.05 | 93.39 | 96.48 | 46.40 | 67 |
| Moslemi Haghighi 2021 | 94.90 | 93.21 | 96.37 | 47.50 | 67 |
| Ke 2020 | 95.03 | 93.36 | 96.47 | 46.80 | 67 |
| Ke 2020 | 94.98 | 93.29 | 96.43 | 47.40 | 67 |
| Shaheiwola 2018 | 94.88 | 93.18 | 96.35 | 47.30 | 67 |
| Ni 2023 | 94.87 | 93.17 | 96.34 | 47.20 | 67 |
| Chen 2025 | 94.84 | 93.14 | 96.31 | 46.80 | 67 |
| Wang 2023 | 94.85 | 93.14 | 96.32 | 46.90 | 67 |

**Supplementary Table S6. (Continued)**

| **Excluded_Study** | **Pooled_Rate** | **CI_Lower** | **CI_Upper** | **I2** | **k** |
| --- | --- | --- | --- | --- | --- |
| Wu 2023 | 94.88 | 93.18 | 96.35 | 47.30 | 67 |
| Yang 2021 | 95.04 | 93.37 | 96.47 | 46.70 | 67 |
| Edwards 2018 | 94.90 | 93.19 | 96.38 | 47.50 | 67 |
| Palimeris 2022 | 94.78 | 93.08 | 96.25 | 45.50 | 67 |
| Liu 2025 | 94.92 | 93.21 | 96.39 | 47.50 | 67 |
| Chen 2021 | 95.00 | 93.32 | 96.46 | 47.20 | 67 |
| Gao 2025 | 95.01 | 93.33 | 96.46 | 47.10 | 67 |
| Tang 2024 | 94.88 | 93.18 | 96.35 | 47.30 | 67 |
| Tang 2024 | 94.98 | 93.30 | 96.44 | 47.40 | 67 |
| Morone 2022 | 94.82 | 93.11 | 96.29 | 46.40 | 67 |
| Lee SH 2025 | 95.04 | 93.37 | 96.47 | 46.70 | 67 |
| Chen 2019 | 94.99 | 93.31 | 96.44 | 47.30 | 67 |
| Salazar 2020 | 94.88 | 93.18 | 96.35 | 47.30 | 67 |
| Pinto 2021 | 94.98 | 93.29 | 96.44 | 47.40 | 67 |
| Zheng 2015 | 94.96 | 93.24 | 96.43 | 47.50 | 67 |
| Zhao 2022 | 94.82 | 93.12 | 96.30 | 46.60 | 67 |
| Yao 2020 | 95.01 | 93.33 | 96.46 | 47.10 | 67 |

*Note: Each row represents the pooled completion rate after excluding the specified study. k = number of remaining comparisons after exclusion.*

**Supplementary Table S7. Cumulative meta-analysis by publication year**

| **Year** | **Cumulative_Rate** | **CI_Lower** | **CI_Upper** |
| --- | --- | --- | --- |
| 2010 | 98.73 | 92.90 | 99.80 |
| 2010 | 98.76 | 94.40 | 99.98 |
| 2011 | 97.85 | 93.91 | 99.80 |
| 2011 | 97.33 | 93.79 | 99.42 |
| 2012 | 97.57 | 94.45 | 99.44 |
| 2012 | 97.66 | 94.77 | 99.42 |
| 2013 | 97.78 | 95.12 | 99.42 |
| 2014 | 97.77 | 95.20 | 99.38 |
| 2014 | 97.20 | 94.47 | 99.02 |
| 2014 | 97.29 | 94.72 | 99.03 |
| 2014 | 97.41 | 94.99 | 99.05 |
| 2015 | 97.55 | 95.30 | 99.08 |
| 2015 | 97.02 | 94.85 | 98.61 |
| 2015 | 96.79 | 94.61 | 98.42 |
| 2015 | 96.94 | 94.87 | 98.49 |
| 2016 | 97 | 94.99 | 98.51 |
| 2016 | 96.68 | 94.62 | 98.26 |
| 2016 | 96.73 | 94.72 | 98.27 |
| 2016 | 96.42 | 94.39 | 98.01 |
| 2017 | 95.49 | 92.43 | 97.79 |
| 2018 | 95.64 | 92.74 | 97.83 |
| 2018 | 95.70 | 93.02 | 97.76 |
| 2018 | 95.08 | 92.01 | 97.43 |
| 2018 | 94.24 | 90.58 | 97.04 |
| 2018 | 94.21 | 90.92 | 96.80 |
| 2018 | 94.46 | 91.31 | 96.93 |
| 2018 | 94.67 | 91.66 | 97.04 |

**Supplementary Table S7. (Continued)**

| **Year** | **Cumulative_Rate** | **CI_Lower** | **CI_Upper** |
| --- | --- | --- | --- |
| 2019 | 94.54 | 91.58 | 96.90 |
| 2019 | 94.77 | 91.93 | 97.03 |
| 2019 | 94.95 | 92.22 | 97.12 |
| 2019 | 95.11 | 92.48 | 97.20 |
| 2020 | 95.02 | 92.45 | 97.09 |
| 2020 | 94.83 | 92.29 | 96.89 |
| 2020 | 94.94 | 92.48 | 96.94 |
| 2020 | 95.05 | 92.66 | 96.99 |
| 2020 | 95.15 | 92.83 | 97.03 |
| 2020 | 94.97 | 92.65 | 96.88 |
| 2020 | 94.84 | 92.55 | 96.74 |
| 2020 | 94.95 | 92.71 | 96.79 |
| 2021 | 94.97 | 92.81 | 96.76 |
| 2021 | 94.90 | 92.79 | 96.66 |
| 2021 | 94.55 | 92.32 | 96.42 |
| 2021 | 94.22 | 91.89 | 96.18 |
| 2021 | 94.28 | 92 | 96.21 |
| 2021 | 94.19 | 91.94 | 96.10 |
| 2021 | 94.05 | 91.79 | 95.96 |
| 2022 | 94.24 | 92.04 | 96.11 |
| 2022 | 94.47 | 92.31 | 96.29 |
| 2022 | 94.55 | 92.44 | 96.34 |
| 2022 | 94.70 | 92.64 | 96.44 |
| 2022 | 94.77 | 92.75 | 96.48 |
| 2023 | 94.89 | 92.91 | 96.56 |
| 2023 | 94.74 | 92.75 | 96.44 |
| 2023 | 94.79 | 92.83 | 96.46 |

**Supplementary Table S7. (Continued)**

| **Year** | **Cumulative_Rate** | **CI_Lower** | **CI_Upper** |
| --- | --- | --- | --- |
| 2023 | 94.85 | 92.92 | 96.49 |
| 2023 | 94.91 | 93.02 | 96.52 |
| 2023 | 94.75 | 92.83 | 96.38 |
| 2023 | 94.83 | 92.95 | 96.44 |
| 2023 | 94.90 | 93.05 | 96.48 |
| 2024 | 94.85 | 93.02 | 96.42 |
| 2024 | 94.91 | 93.11 | 96.45 |
| 2025 | 94.83 | 93.04 | 96.37 |
| 2025 | 94.93 | 93.18 | 96.44 |
| 2025 | 94.83 | 93.07 | 96.34 |
| 2025 | 94.87 | 93.14 | 96.37 |
| 2025 | 94.92 | 93.21 | 96.39 |
| 2025 | 94.94 | 93.26 | 96.38 |

**Supplementary Table S8. League table of pairwise relative risks (NMA)**

| **Unnamed: 0** | **Sham/Control** | **A-tDCS** | **BL-TBS** | **BL-rTMS** | **BL-tDCS** | **C-tDCS** | **Combined Protocol** | **Dual-tDCS** | **HD-tDCS** | **HF-rTMS** | **LF-rTMS** | **Non-priming iTBS** | **Priming iTBS** | **cTBS** | **iTBS** |
| --- | --- | --- | --- | --- | --- | --- | --- | --- | --- | --- | --- | --- | --- | --- | --- |
| Sham/Control | Sham/Control | 0.99 (0.97-1.02) | 1 (0.91-1.10) | 0.99 (0.93-1.05) | 1 (0.91-1.10) | 1.05 (0.97-1.13) | 1 (0.92-1.09) | 0.99 (0.96-1.03) | 1 (0.74-1.35) | 1.01 (0.97-1.06) | 1.01 (0.98-1.04) | 1 (0.88-1.14) | 1 (0.88-1.14) | 1.02 (0.86-1.21) | 0.97 (0.91-1.04) |
| A-tDCS | 1.01 (0.98-1.03) | A-tDCS | 1.01 (0.91-1.11) | 0.99 (0.92-1.07) | 1.01 (0.91-1.12) | 1.05 (0.97-1.14) | 1.01 (0.92-1.10) | 1 (0.95-1.05) | 1.01 (0.74-1.36) | 1.02 (0.96-1.08) | 1.01 (0.97-1.06) | 1.01 (0.88-1.15) | 1.01 (0.88-1.15) | 1.03 (0.87-1.22) | 0.98 (0.91-1.06) |
| BL-TBS | 1 (0.91-1.10) | 0.99 (0.90-1.10) | BL-TBS | 0.99 (0.88-1.11) | 1 (0.87-1.15) | 1.05 (0.93-1.18) | 1 (0.88-1.13) | 0.99 (0.90-1.10) | 1 (0.73-1.37) | 1.01 (0.91-1.13) | 1.01 (0.91-1.11) | 1 (0.85-1.18) | 1 (0.85-1.18) | 1.02 (0.84-1.25) | 0.97 (0.87-1.10) |
| BL-rTMS | 1.01 (0.95-1.08) | 1.01 (0.94-1.08) | 1.01 (0.90-1.14) | BL-rTMS | 1.01 (0.90-1.14) | 1.06 (0.96-1.17) | 1.01 (0.91-1.12) | 1.01 (0.93-1.08) | 1.01 (0.74-1.38) | 1.03 (0.95-1.11) | 1.02 (0.95-1.10) | 1.01 (0.87-1.17) | 1.01 (0.87-1.17) | 1.04 (0.86-1.24) | 0.99 (0.90-1.08) |
| BL-tDCS | 1 (0.91-1.10) | 0.99 (0.90-1.10) | 1 (0.87-1.15) | 0.99 (0.88-1.11) | BL-tDCS | 1.05 (0.92-1.19) | 1 (0.88-1.14) | 0.99 (0.89-1.10) | 1 (0.73-1.38) | 1.01 (0.91-1.13) | 1.01 (0.91-1.12) | 1 (0.85-1.18) | 1 (0.85-1.18) | 1.02 (0.84-1.25) | 0.97 (0.86-1.10) |
| C-tDCS | 0.96 (0.89-1.03) | 0.95 (0.88-1.03) | 0.96 (0.85-1.08) | 0.94 (0.85-1.04) | 0.96 (0.84-1.08) | C-tDCS | 0.96 (0.85-1.07) | 0.95 (0.87-1.03) | 0.96 (0.70-1.31) | 0.97 (0.89-1.06) | 0.96 (0.89-1.04) | 0.96 (0.82-1.11) | 0.96 (0.82-1.11) | 0.98 (0.81-1.18) | 0.93 (0.84-1.03) |
| Combined Protocol | 1 (0.92-1.09) | 0.99 (0.91-1.09) | 1 (0.88-1.13) | 0.99 (0.89-1.10) | 1 (0.88-1.14) | 1.05 (0.94-1.17) | Combined Protocol | 0.99 (0.91-1.09) | 1 (0.73-1.37) | 1.01 (0.92-1.11) | 1.01 (0.92-1.10) | 1 (0.86-1.17) | 1 (0.86-1.17) | 1.02 (0.85-1.24) | 0.97 (0.87-1.09) |
| Dual-tDCS | 1.01 (0.97-1.04) | 1 (0.96-1.05) | 1.01 (0.91-1.11) | 0.99 (0.92-1.07) | 1.01 (0.91-1.12) | 1.05 (0.97-1.14) | 1.01 (0.92-1.10) | Dual-tDCS | 1.01 (0.74-1.36) | 1.02 (0.96-1.08) | 1.02 (0.97-1.06) | 1.01 (0.88-1.15) | 1.01 (0.88-1.15) | 1.03 (0.87-1.23) | 0.98 (0.91-1.06) |
| HD-tDCS | 1 (0.74-1.35) | 0.99 (0.73-1.35) | 1 (0.73-1.37) | 0.99 (0.72-1.35) | 1 (0.73-1.38) | 1.05 (0.77-1.43) | 1 (0.73-1.37) | 0.99 (0.73-1.35) | HD-tDCS | 1.01 (0.75-1.38) | 1.01 (0.74-1.37) | 1 (0.72-1.39) | 1 (0.72-1.39) | 1.02 (0.72-1.45) | 0.97 (0.71-1.33) |
| HF-rTMS | 0.99 (0.94-1.03) | 0.98 (0.93-1.04) | 0.99 (0.89-1.10) | 0.97 (0.90-1.06) | 0.99 (0.88-1.10) | 1.03 (0.95-1.13) | 0.99 (0.90-1.08) | 0.98 (0.93-1.04) | 0.99 (0.73-1.34) | HF-rTMS | 1 (0.94-1.05) | 0.99 (0.86-1.14) | 0.99 (0.86-1.14) | 1.01 (0.85-1.21) | 0.96 (0.88-1.04) |
| LF-rTMS | 0.99 (0.96-1.02) | 0.99 (0.95-1.03) | 0.99 (0.90-1.09) | 0.98 (0.91-1.05) | 0.99 (0.89-1.10) | 1.04 (0.96-1.12) | 0.99 (0.91-1.08) | 0.99 (0.94-1.03) | 0.99 (0.73-1.34) | 1 (0.95-1.06) | LF-rTMS | 0.99 (0.87-1.14) | 0.99 (0.87-1.14) | 1.02 (0.86-1.21) | 0.97 (0.90-1.04) |
| Non-priming iTBS | 1 (0.88-1.14) | 0.99 (0.87-1.14) | 1 (0.85-1.18) | 0.99 (0.85-1.15) | 1 (0.85-1.18) | 1.05 (0.90-1.22) | 1 (0.86-1.17) | 0.99 (0.87-1.14) | 1 (0.72-1.39) | 1.01 (0.88-1.17) | 1.01 (0.88-1.16) | Non-priming iTBS | 1 (0.83-1.21) | 1.02 (0.83-1.27) | 0.97 (0.84-1.13) |
| Priming iTBS | 1 (0.88-1.14) | 0.99 (0.87-1.14) | 1 (0.85-1.18) | 0.99 (0.85-1.15) | 1 (0.85-1.18) | 1.05 (0.90-1.22) | 1 (0.86-1.17) | 0.99 (0.87-1.14) | 1 (0.72-1.39) | 1.01 (0.88-1.17) | 1.01 (0.88-1.16) | 1 (0.83-1.21) | Priming iTBS | 1.02 (0.83-1.27) | 0.97 (0.84-1.13) |
| cTBS | 0.98 (0.82-1.16) | 0.97 (0.82-1.15) | 0.98 (0.80-1.19) | 0.96 (0.80-1.16) | 0.98 (0.80-1.19) | 1.02 (0.85-1.23) | 0.98 (0.81-1.18) | 0.97 (0.81-1.15) | 0.98 (0.69-1.38) | 0.99 (0.83-1.18) | 0.98 (0.83-1.17) | 0.98 (0.79-1.21) | 0.98 (0.79-1.21) | cTBS | 0.95 (0.79-1.14) |
| iTBS | 1.03 (0.96-1.10) | 1.02 (0.95-1.10) | 1.03 (0.91-1.16) | 1.01 (0.92-1.12) | 1.03 (0.91-1.16) | 1.07 (0.97-1.19) | 1.03 (0.92-1.15) | 1.02 (0.94-1.10) | 1.03 (0.75-1.40) | 1.04 (0.96-1.13) | 1.04 (0.96-1.12) | 1.03 (0.88-1.19) | 1.03 (0.88-1.19) | 1.05 (0.88-1.27) | iTBS |

*Note: Upper triangle (above diagonal): RR for row vs column treatment. Lower triangle (below diagonal): RR for column vs row treatment. All 95% CrIs include 1.00, indicating no statistically significant differences.*

**Supplementary Table S9. Meta-regression (univariate)**

| **Predictor** | **Coefficient** | **SE** | **P_value** | **R_squared** | **N** |
| --- | --- | --- | --- | --- | --- |
| pub_year | -0.10 | 0.26 | 0.71 | 0 | 68 |
| int_enrolled | -0.01 | 0.07 | 0.93 | 0 | 68 |
| planned_sessions | 0.12 | 0.16 | 0.46 | 0.01 | 68 |

**Supplementary Table S10. Adverse events by type**

| **AE_type** | **n_studies** | **int_events** | **ctrl_events** |
| --- | --- | --- | --- |
| Skin tingling | 12 | 64 | 32 |
| Headache | 12 | 63 | 15 |
| Skin redness | 4 | 32 | 1 |
| Epileptiform EEG | 1 | 29 | 0 |
| Scalp discomfort | 4 | 20 | 5 |
| Itching | 3 | 19 | 12 |
| Drowsiness | 1 | 13 | 11 |
| Facial twitch | 1 | 3 | 0 |
| Neck pain | 1 | 3 | 1 |
| Skin redness/itching | 1 | 3 | 2 |
| Dizziness | 1 | 2 | 1 |
| Fatigue | 2 | 2 | 0 |
| Scalp numbness | 1 | 2 | 0 |
| Seizure | 1 | 2 | 0 |
| Nausea | 1 | 1 | 0 |
| Skin blister | 1 | 1 | 0 |

**Supplementary Table S11. Serious adverse events**

| **AE_type** | **n_studies** | **int_n** | **ctrl_n** | **total** |
| --- | --- | --- | --- | --- |
| Seizure | 1 | 2 | 0 | 2 |

**Supplementary Table S12. Studies by country**

| **Country** | **k** | **Pooled_Rate** | **CI_Lower** | **CI_Upper** |
| --- | --- | --- | --- | --- |
| China | 26 | 94.10 | 90.90 | 96.70 |
| Taiwan | 7 | 97.70 | 94 | 99.70 |
| Italy | 5 | 97.40 | 92 | 99.90 |
| South Korea | 4 | 89.60 | 79.80 | 96.40 |
| USA | 4 | 91.90 | 84.90 | 96.90 |
| Egypt | 4 | 98.50 | 94.30 | 100 |
| Germany | 3 | 96.50 | 91.60 | 99.30 |
| Brazil | 3 | 98.20 | 92.10 | 100 |
| India | 2 | 95.70 | 86.60 | 99.80 |
| Japan | 2 | 94.50 | 79.80 | 100 |
| Russia | 2 | 65.90 | 50.10 | 80.10 |
| Iran | 1 | 100 | 72.20 | 100 |
| Poland | 1 | 100 | 83.90 | 100 |
| Serbia | 1 | 100 | 78.50 | 100 |
| Canada | 1 | 100 | 92.60 | 100 |
| UK | 1 | 91.70 | 64.60 | 98.50 |
| Belgium | 1 | 100 | 86.70 | 100 |

**Supplementary Table S13. GRADE evidence quality assessment**

| **Comparison** | **k** | **RR (95% CI)** | **Risk of Bias** | **Inconsistency** | **Indirectness** | **Imprecision** | **Publication Bias** | **Quality** | **Quality_Score** |
| --- | --- | --- | --- | --- | --- | --- | --- | --- | --- |
| A-tDCS vs Sham/Control | 12 | 1 (0.98-1.03) | -1 | 0 | 0 | 0 | 0 | Moderate | 3 |
| BL-TBS vs Sham/Control | 1 | 1 (0.91-1.10) | -1 | 0 | 0 | 0 | -1 | Low | 2 |
| BL-rTMS vs Sham/Control | 3 | 1.01 (0.95-1.08) | -1 | 0 | 0 | 0 | -1 | Low | 2 |
| BL-tDCS vs Sham/Control | 2 | 1 (0.91-1.10) | -1 | 0 | 0 | 0 | -1 | Low | 2 |
| C-tDCS vs Sham/Control | 2 | 0.96 (0.89-1.03) | -1 | 0 | 0 | 0 | -1 | Low | 2 |
| Combined Protocol vs Sham/Control | 2 | 1 (0.92-1.09) | -1 | 0 | 0 | 0 | -1 | Low | 2 |
| Dual-tDCS vs Sham/Control | 8 | 1.01 (0.97-1.04) | -1 | 0 | 0 | 0 | -1 | Low | 2 |
| HD-tDCS vs Sham/Control | 1 | 1 (0.74-1.35) | -1 | 0 | 0 | -1 | -1 | Very Low | 1 |
| HF-rTMS vs Sham/Control | 14 | 0.99 (0.94-1.03) | -1 | 0 | 0 | 0 | 0 | Moderate | 3 |
| LF-rTMS vs Sham/Control | 15 | 0.99 (0.96-1.02) | -1 | 0 | 0 | 0 | 0 | Moderate | 3 |
| Non-priming iTBS vs Sham/Control | 1 | 1 (0.88-1.14) | -1 | 0 | 0 | 0 | -1 | Low | 2 |
| Priming iTBS vs Sham/Control | 1 | 1 (0.88-1.14) | -1 | 0 | 0 | 0 | -1 | Low | 2 |
| cTBS vs Sham/Control | 2 | 0.98 (0.82-1.16) | -1 | 0 | 0 | 0 | -1 | Low | 2 |
| iTBS vs Sham/Control | 4 | 1.03 (0.96-1.10) | -1 | 0 | 0 | 0 | -1 | Low | 2 |

*Note: GRADE = Grading of Recommendations, Assessment, Development and Evaluations. Downgrading criteria: Risk of Bias (-1 = serious concerns); Imprecision (-1 = wide CIs); Publication Bias (-1 = suspected). Quality: High (4), Moderate (3), Low (2), Very Low (1).*

**Supplementary Figure S1.** Network meta-analysis and safety details
(A) Rankogram showing the probability of each treatment ranking at each position based on Bayesian simulations. (B) Pairwise RR comparison matrix (heatmap). Green=RR>1 (row treatment favoured); red=RR<1. (C) AE rate by study for intervention groups. Bar length represents proportion of participants experiencing any AE.

**Supplementary Figure S2.** Exploratory analyses
(A) Dropout reasons by category in intervention and control groups. (B) Completion rate trends by publication year for intervention (red) and control (blue) groups. (C) Meta-regression of publication year versus completion rate (β=−0.097, P=0.712, R²=0.002).

**Supplementary Figure S3.** Sensitivity analysis and publication bias
(A) Leave-one-out analysis. Pooled rate ranged from 94.78% to 95.23% across iterations, indicating high stability. Dashed line=overall estimate; shaded area=95% CI. (B) Doi plot for publication bias. LFK index=0.04 (no asymmetry). (C) Cumulative meta-analysis by publication year. Estimate stabilised from approximately 2015.
